# Double the Chemistry, Double the Fun: Structural Diversity and Biological Activity of Marine-Derived Diketopiperazine Dimers

**DOI:** 10.3390/md17100551

**Published:** 2019-09-27

**Authors:** Nelson G. M. Gomes, Renato B. Pereira, Paula B. Andrade, Patrícia Valentão

**Affiliations:** REQUIMTE/LAQV, Laboratório de Farmacognosia, Departamento de Química, Faculdade de Farmácia, Universidade do Porto, R. Jorge Viterbo Ferreira, nº 228, Porto 4050-313, Portugal; ngomes@ff.up.pt (N.G.M.G.); ren.pereira@gmail.com (R.B.P.); pandrade@ff.up.pt (P.B.A.)

**Keywords:** asperdimin, asperflocin, aspergilazine A, brevianamide S, chetracins, cristatumins, cristazine, eurocristatine, leptosins, naseseazines

## Abstract

While several marine natural products bearing the 2,5-diketopiperazine ring have been reported to date, the unique chemistry of dimeric frameworks appears to remain neglected. Frequently reported from marine-derived strains of fungi, many naturally occurring diketopiperazine dimers have been shown to display a wide spectrum of pharmacological properties, particularly within the field of cancer and antimicrobial therapy. While their structures illustrate the unmatched power of marine biosynthetic machinery, often exhibiting unsymmetrical connections with rare linkage frameworks, enhanced binding ability to a variety of pharmacologically relevant receptors has been also witnessed. The existence of a bifunctional linker to anchor two substrates, resulting in a higher concentration of pharmacophores in proximity to recognition sites of several receptors involved in human diseases, portrays this group of metabolites as privileged lead structures for advanced pre-clinical and clinical studies. Despite the structural novelty of various marine diketopiperazine dimers and their relevant bioactive properties in several models of disease, to our knowledge, this attractive subclass of compounds is reviewed here for the first time.

## 1. General Considerations

Bioprospection of marine organisms as producers of structurally complex and biologically active metabolites has been particularly rewarding as there are now over 29,000 marine natural products, approximately 41% being discovered in the last ten years [1]. So far, the clinical utility of marine-derived agents has been translated into the development of seven drugs approved by the Food and Drug Administration (FDA) and the European Medicines Agency (EMA) [2,3]. The discovery of increasing numbers of marine natural products has also spurred the investigation of the medicinal chemistry of diketopiperazines which appear to abound in the marine environment [4,5]. 

As considered in the current review, the conventional definition of “diketopiperazines” almost exclusively deals with 2,5-diketopiperazines; however, it should be noted that two additional types of regioisomers are possible i.e., 2,3 and 2,6-heterocyclic scaffolds [6]. In fact, 2,5-diketopiperazines are the best known, not only due to their widespread distribution in natural sources, but also as a result of their therapeutic utility [7,8]. Long solely perceived as degradation products and protein artifacts [9], diketopiperazines have been used as key structural fragments in drug design, which has been translated into the development of several drugs and an increasing number of clinical candidates, with more than 150 global patents being registered by August 2012 [10]. In fact, their conformationally constrained structural backbone represents a relevant pharmacophore in medicinal chemistry due to its stable structural characteristics [7]. For example, diketopiperazines have found therapeutic application in the treatment of erectile dysfunction as inhibitors of phosphodiesterase-5 [11], as well as antagonists of oxytocin for the treatment of preterm labor [12]. Another significant example is the vascular disrupting and tubulin-depolymerizing agent plinabulin, based on the marine fungal agent halimide, and currently on the last stage of clinical development for the treatment of non-small-cell lung cancer (NSCLC) [13,14,15]. The diketopiperazine-derived template has been also investigated as a brain shuttle for the delivery of medicinal agents with limited ability to cross the blood-brain barrier, bypassing the limited bioavailability of several drugs [16,17]. Additionally, diketopiperazine-containing compounds have also long been used as catalysts and chiral auxiliaries in synthetic organic chemistry [18,19].

The diketopiperazine scaffold occurs in several natural products, frequently embedded in complex metabolic architectures, mostly produced by microorganisms, but also occurring in plants and animals [7,20]. A substantial number of diketopiperazines has been reported from bacteria, but most have been isolated from marine and terrestrial fungi, *Aspergillus* and *Penicillium* species being particularly fruitful sources [21,22,23,24]. In fact, it is noteworthy that tryptophan-containing heterocycles are amongst the most prevalent natural products harboring a diketopiperazine core, the majority of which being isolated from fungal species [22,25].

Diketopiperazines are the smallest of all cyclopeptides and derive almost exclusively from “head-to-tail” cyclization of two amino acids, in which the two nitrogen atoms of the six-membered ring form amide linkages. For many years, biosynthesis of the mainframe structure was thought to be solely catalyzed by the massive multimodular nonribosomal peptide synthetases (NRPSs), long recognized as catalysts for the formation of diketopiperazine scaffolds, particularly in fungi [26,27]. NRPSs act as molecular assembly lines, with each module typically catalyzing a cycle of amino acid adenylation, peptide bond formation, and optional tailoring reactions that further equip the diketopiperazine moiety [28]. Diketopiperazine scaffolds originate predominantly from one or more specialized NRPSs, either through dedicated biosynthetic pathways or through the premature release of dipeptidyl intermediates from longer assembly lines [28]. However, the unrelated and novel enzymatic family of cyclodipeptide synthases (CDPSs), first described in 2009 by Gondry and colleagues [29], was found to be also able to generate the diketopiperazine assembly. In contrast to NRPSs, CDPDs have been mostly characterized from bacterial gene clusters that also encode putative enzymes with hypothesized roles in diketopiperazine tailoring [29,30]. CDPDs divert aminoacyl-tRNAs (aa-tRNAs) from their canonical role in ribosomal translation and recruit them as substrates for the synthesis of diketopiperazines, bridging primary and secondary metabolic pathways [29,31]. The formation of the diketopiperazine ring occurs via a sequential ping-pong mechanism, utilizing two aa-tRNAs as substrates that bind at separate sites of CDPDs, without requiring amino acid charging [32,33]. 

The number of elucidated biosynthetic routes for the formation of diketopiperazines is notoriously low relative to their structural diversity. So far, ten pathways involving NRPSs have been decoded and only six are known to depend on CDPSs [34]. In fact, putative tailoring enzymes that modify the initially assembled diketopiperazine scaffold can be found in almost all NRPS and CDPS gene clusters, installing a diverse range of structural functionalities [35]. Those include oxidoreductases that modulate the oxidation state of the side chains, prenyltransferases with distinct chemo- and regiospecificities, and cytochromes P450 responsible for the dimerization of diketopiperazine monomers [35,36]. 

Dimeric diketopiperazines are a relevant variation of this class of natural products occupying an enormous chemical complexity, the vast majority of which having a 3α,3α’-bispyrrolidinoindoline core. In fact, tryptophan residues that are part of dimeric diketopiperazines mostly occur in an annulated form, in which an additional five-membered ring is generated through formation between the α-nitrogen and the indole C2 of the tryptophanyl residue, resulting in a tetracyclic ring system [37]. 

As several members of this subclass oftentimes carry biological activities, frequently translated in pharmacological utility, dimeric diketopiperazines have drawn great deal of attention, particularly in the area of medicinal chemistry. However, previous reviews have been predominantly concerned with the occurrence of monomeric diketopiperazines and their bioactivities [8,20,21,24,38,39]. The seminal paper by Borthwick [7] covered the structure, synthesis, biological properties and potential therapeutic applications of diketopiperazines in detail. It is also worth to mention the review by Wang et al. [10] on filed patents assigned to diketopiperazines and their derivatives. Earlier reviews by Huang and colleagues provided general insights into chemical aspects of marine-derived diketopiperazines and their ability to interact with therapeutically relevant targets [4,5].

Reports on dimeric natural products were scarce before the 1960s, as their identification was extremely challenging, displaying spectroscopic data usually identical with their corresponding monomers. The remarkable technological progress in analytical instrumentation allowed both a wider coverage of the metabolome and the detection of new metabolites, along with an increased accuracy in their structure elucidation [40], which has been translated into the discovery of more than 600 dimeric natural products until 2006 [41]. Despite the increasing number of reports on marine dimeric diketopiperazines, no detailed and comprehensive summary on their occurrence, structural diversity and biological properties has been reported. 

## 2. Chemistry and Biological Properties of Marine Diketopiperazine Dimers 

Dimeric diketopiperazines encompass a complex structural architecture with a densely functionalized core structure with many stereogenic centers. Most diketopiperazine dimers possess vicinal quaternary stereocenters joined through sterically congested C3(sp^3^)-C3’(sp^3^) bonds, forming a characteristic arrangement of two contiguous quaternary stereogenic centers with the same configuration (Section 2.1). However, monomeric units can be also connected through unusual linkages as through C3(sp^3^)-C7’(sp^2^) and C3(sp^3^)-C6’(sp^2^) bridges, and between the C3(sp^3^) of one hexahydropyrroloindole unit and N1’ from tryptophan from an analogous diketopiperazine unit containing an extra indole (Section 2.2).

### 2.1. Dimers Joined Through C3-C3’ Bond

**Figure 1:**
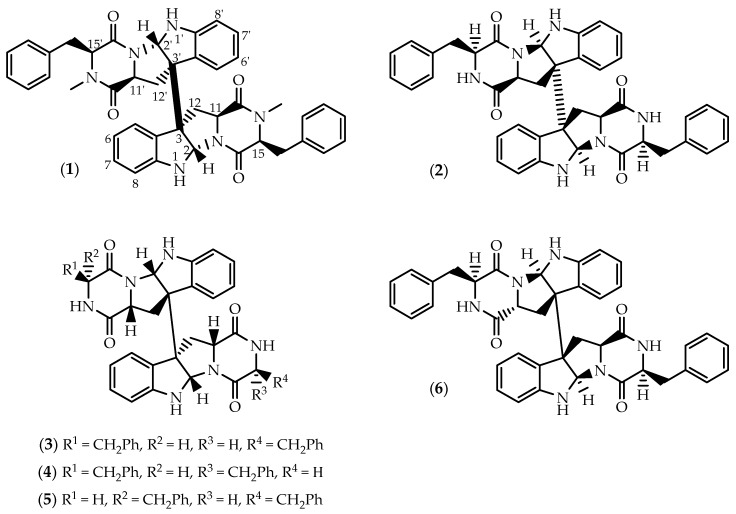
Structures of ditryptophenaline (**1**), WIN 64821 (**2**) and asymmetric stereoisomers (**3**–**6**).

Ditryptophenaline (**1**) (Figure 1) was first isolated from the mycelium of several strains of *Aspergillus flavus* obtained from contaminated food [42]. Later, the homodimeric diketopiperazine was reported from two marine-derived strains of *A. flavus*, isolated from the alga *Enteromorpha tubulosa* [43] and from the prawn *Penaeus vannamei* [44], as well as from an endophytic *Penicillium cyclopium* obtained from the mangrove plant *Salicornia bigelovii* [45]. 

From a single crystal X-ray experiment, Springer and colleagues assigned the gross structure of **1** and its relative configuration, being the first structurally characterized dimeric diketopiperazine [42,46]. The first total synthesis of (–)-ditryptophenaline (**1**) was achieved in 1981, through a biomimetic thallium(III)-promoted oxidative dimerization allowing the determination of its absolute stereochemistry [46]. The *S* configuration at the ring junctions C2/C2’ and C3/C3’, and the two *N*-methyl-L-phenylalanines involved in the dimeric moiety, were later corroborated by Maes et al. [47], through additional NMR assignments and circular dichroism experiments. Relevantly, the early work by Nakagawa and colleagues [46] provided preliminary but relevant clues on the biosynthetic pathway involved in the production of **1**, which was further elucidated by Saruwatari and co-workers [48], suggesting that the cytochrome P450 DtpC is responsible for both pyrroloindole ring formation and the concomitant dimerization through a radical-mediated coupling reaction.

While ditryptophenaline (**1**) was reported to be a weak competitive antagonist of substance P at the human neurokinin-1 (NK-1) receptor in human U-373 MG astrocytoma cells [49], the related dimer WIN 64821 (**2**) (Figure 1) exhibited submicromolar potency [50,51]. WIN 64821 (**2**) was first isolated from an EtOAc extract of whole fermentation broths of a soil-derived *Aspergillus* sp. (SC319, ATCC 74177) at Sterling Winthrop Pharmaceuticals by the group of Colin Barrow [50,51]. The homodimer was later isolated from cultures of a strain of *Aspergillus* sp. obtained from samples of the Rhodophyta *Porphyra yezoensis*, being found to exhibit moderate cytotoxicity against 37 human tumor cell lines [52]. Despite the cytotoxic effects upon several cancer cell lines, WIN 64821 (**2**) is mainly reputed due to its potent effects against the endogenous ligand of the human NK-1 receptor, which sparked the development of several analogues through directed biosynthesis [53,54]. Closely related to (–)-ditryptophenaline (**1**), (+)-WIN 64821 (**2**) presents an opposite stereochemistry at the indoline bridge and methyl substitutions at the secondary amines, the *R* chirality at the ring junctions being a structural determinant for NK-1 binding [49,50,53]. Structure-activity relationship (SAR) studies also suggested the involvement of both the indoline and phenyl moieties in substance P receptor binding [53,54]. Additionally, WIN 64821 (**2**) demonstrated equipotent activity and functional antagonism against neurokinin-2 (NK-2) receptor, being also reported as an antagonist of the cholecystokinin type-B (CCK-B) receptor [55]. In 2001, Overman and Paone [56] described the enantioselective total synthesis of *ent*-WIN 64821 confirming the structure proposed for **2**.

WIN 64821 (**2**) was isolated along with a series of stereoisomers displaying structural asymmetry (**3**–**6**) (Figure 1) from extracts of marine-derived *Aspergillus* sp. Chemical investigation of cultures of *Aspergillus* sp. DX4H obtained from shrimp collected in seaside of Dinghai (China), afforded the diastereomers **3**–**5**, containing a different configuration at C2, C2’ and C3’ from those in WIN 64821 (**2**) (Figure 1) [57]. Weak in vitro cytotoxicity was recorded towards prostate cancer PC3 cells upon exposure to **2**–**5** at 30 μM [57]. Recently, the epimer asperflocin (**6**) (Figure 1) was obtained from an EtOAc extract of *A. versicolor* 16F-11 isolated from the marine sponge *Phakellia fusca*, collected from Yongxing Island in China [58]. Solely differing from **2** on the chirality of C11’, asperflocin (**6**) was possibly biosynthesized by fungus through the random selection of L- or D-tryptophan since different residues are embedded in the structure [58]. Despite being assayed against a panel of four cancer cell lines, **6** only proved to exert cytotoxicity against human melanoma A375 cells [58].

**Figure 2:**
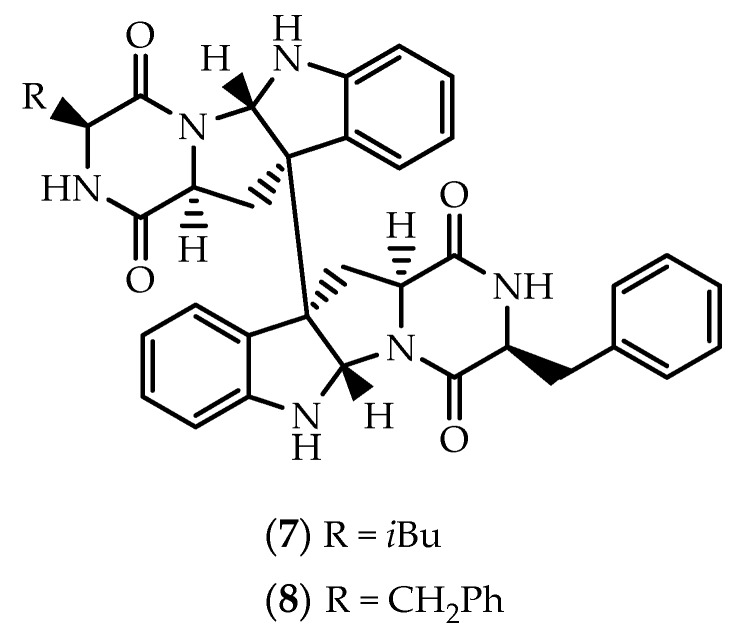
Structures of SF5280-451 (**7**) and SF5280-415 (**8**).

The strain *Aspergillus* sp. SF-5280, isolated from sponge material collected at Cheju Island in Korea, was found to produce SF5280-451(**7**) and SF5280-415 (**8**) (Figure 2) [59]. First reported in 2015 from a fungal isolate of *Aspergillus sydowii* (MSX19583) obtained from spruce litter [60], SF5280 (**7**) is a symmetric diastereomer of WIN 64821 (**2**), displaying an opposite stereochemistry at the ring junctions. Both **7** and **8** exhibited inhibitory effects against the activity of protein tyrosine phosphatase 1B (PTP1B), with IC_50_ values of 12.9 and 14.2 μM, respectively [59].

**Figure 3:**
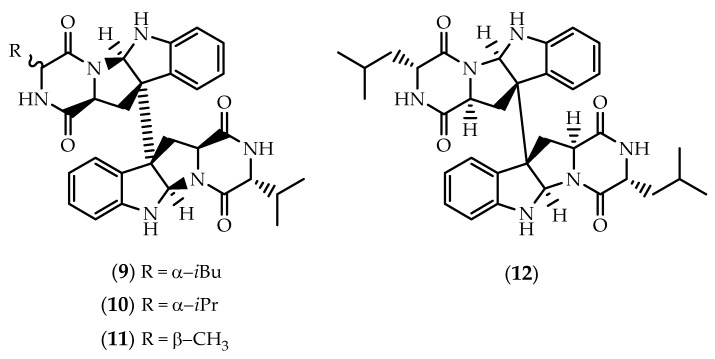
Structures of asperdimin (**9**), eurocristatine (**10**), cristatumin C (**11**) and (**12**).

During a screening for inhibitors of viral internal ribosomal entry site (IRES), a MeOH extract obtained from a marine-derived isolate of *Aspergillus niger* yielded asperdimin (**9**) (Figure 3) [61]. Determination of the absolute configuration was initially attempted by chiral HPLC analysis [61], but structural revision of **9** was later attained by de Lera and collaborators through an elegant stereocontrolled total synthetic approach [62].

Eurocristatine (**10**) (Figure 3) was first reported by Gomes et al. [63], from an EtOAc extract obtained from cultures of the fungus *Eurotium cristatum* KUFC 7356, isolated from a marine sponge *Mycale* sp. collected in Wonnapa beach, Chonburi Province, Thailand. Final proof of the structure and stereochemistry assigned to **10** was provided by X-ray analysis [63]. The homodimeric and bilaterally symmetric dimer was also obtained from cultures of the algal fungus *Eurotium herbariorum* HT-2 [64], and from cultures of *Eurotium chevalieri* KUFA 0006 isolated from the mangrove plant *Rhizophora mucronata* [65]. While proving to be ineffective against MCF-7, NCI-H-460 and A375-C5 cells [63], eurocristatine (**10**) has been found by others to display in vitro cytotoxicity against the K562 chronic myeloid leukemia cell line (IC_50_ = 8.3 µM), as well as weak antibacterial effects towards *Enterobacter aerogenes* and *Escherichia coli* [64]. (+)-Eurocristatine (**10**) and the 15,15’ bis-epimer were later synthesized through a concise two one-pot procedure, leading to the discovery of a new chemotherapeutic candidate through ubiquitin-specific protease 7 (USP7) inhibition [66].

Spectroscopic data of cristatumin C (**11**) (Figure 3) evidenced that one D-valine unit in eurocristatine (**10**) was replaced by an L-alanine residue [67]. Cristatumin C (**11**) was identified from the culture EtOAc extract of *Eurotium cristatum* EN-220, isolated from the marine alga *Sargassum thunbergii* [67], but its structure was corrected by total synthesis of a rationally guided stereoisomer [68].

Chemical investigation of a strain of *Aspergillus violaceofuscus*, isolated from the inner part of a marine sponge *Reniochalina* sp. collected from the Xisha Islands in the South China Sea, afforded three cyclic peptides, including the symmetric homodimer **12** (Figure 3) [69]. Marfey’s analysis of **12** suggested that the stereochemistry at the ring junctions was the same as in **9**–**11** [69]. LPS-challenged THP-1 cells pre-treated with **12** (10 µM) exhibited a significant decrease in IL-10 levels, with an inhibitory rate of 78.1%, thus suggesting a potential anti-inflammatory effect [69].

Epithiodiketopiperazines correspond to an intriguing subclass of metabolites which are generally characterized by sensitive (poly)sulfide bridges spanning the α-carbons of the diketopiperazine ring. Since the seminal discovery of the antibacterial gliotoxin more than 80 years ago [70,71], more than 100 epithiodiketopiperazines have been reported from fungi and lichens [72,73]. Most commonly, the cyclic sulfide functionality is characterized by a disulfide bridge, but several sulfides containing one, three or four sulfur atoms, have been also reported [72,73]. Epithiodiketopiperazines have been globally reputed due to their toxicity, predominantly deriving from the presence of the sulfide bridge that can inactivate thiol-containing proteins, and due to the generation of reactive oxygen species (ROS) by redox cycling. Readers are referred to the reviews from Waring [74,75] and references therein for further details on the general toxicity of epithiodiketopiperazines. Despite their intrinsic toxicity, several epithiodiketopiperazines have proved to exert relevant biological and pharmacological effects [75,76,77]. So far, 35 dimeric epithiodiketopiperazines have been reported from marine sources, frequently characterized by elegant structural backbones and also displaying a wide spectrum of biological properties.

**Figure 4:**
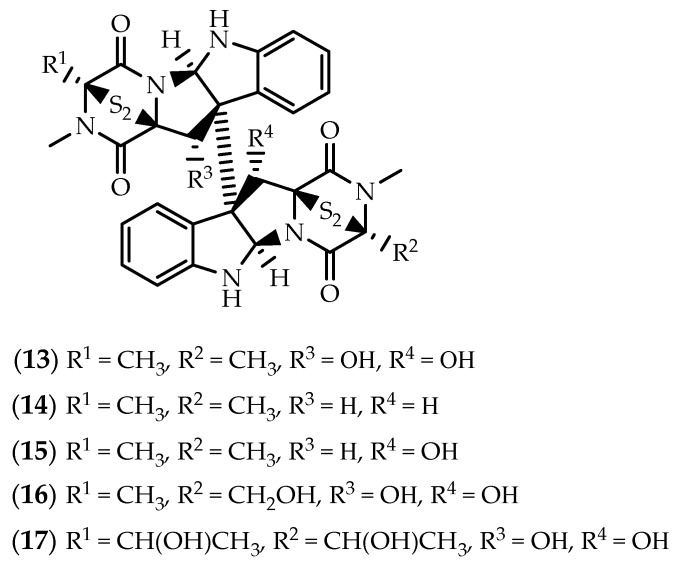
Structures of verticillin A (**13**), 11,11’-dideoxyverticillin A (**14**), 11’-deoxyverticillin A (**15**) and verticillins B-C (**16**–**17**).

In 1999, Fenical’s group described the purification of three dimers encompassing a disulfide-bridged diketopiperazine system (**13**–**15**) (Figure 4), from a marine-derived fungi *Penicillium* sp., obtained from the surface of the Caribbean Chlorophyta *Avrainvillea longicaulis* [78]. Verticillin A (**13**), 11,11’-dideoxyverticillin A (12,12’-dideoxyverticillin A) (**14**) and 11’-deoxyverticillin A (12’-deoxyverticillin A) (**15**) were described as potent cytotoxic metabolites against human HCT-116 colon carcinoma cells, with IC_50_ values in the low nanogram range [78]. In another study, both **13** and **15** displayed cytotoxicity in a panel of human cancer cell lines, with IC_50_ values ranging from 20 to 370 nM, approaching the activity of the anticancer drug camptothecin [79]. Verticillin A (**13**) and 11’-deoxyverticillin A (**15**) were also found to be effective nematicidal agents towards *Caenorhabditis elegans* and *Panagrellus redivivus* [80].

Despite the previous studies, and while sharing close structural similarity with **14** and **15**, verticillin A (**13**) displays very different biological effects. Verticillin A (**13**) was discovered as an antibacterial agent in 1970, and reported as a metabolic product of the *Verticillium* sp. strain TM-759, an imperfect fungus isolated from a basidiocarp of *Coltricia cinnamomea* [81,82]. Since the preliminary study on its in vivo antitumor activity in an Ehrlich ascites carcinoma mice model [81], **13** have drawn great interest of cancer researchers. Verticillin A (**13**) demonstrated anticancer activity via chromatin remodeling, proving to be a potential candidate in overcoming colon carcinoma and pancreatic ductal adenocarcinoma (PDAC) cell resistance, mainly due to the selective inhibition of the histone methyltransferases (HMTases) SUV39H1, SUV39H2, G9a, GLP, NSD2 and MLL1 [83,84]. Early reports indicated that **13** inhibits *c-fos* proto-oncogene induction, suggesting that it could be acting at a very early step, responsible for activation of multiple signaling pathways involved in cell proliferation [85]. Years later, Figueroa et al. [79] described that verticillin A (**13**) was able to inhibit the specific binding ability of activated p65 subunits of NF-kB in the nucleus of HeLa cells with an IC_50_ value as low as 0.1 µM, providing preliminary evidence that **13** was able to interfere with the regulation of programmed cell death.

However, verticillin A (**13**) was brought into the spotlight due to the ability to suppress human colon carcinoma cells immune escape and to overcome 5-fluorouracil (5-FU) chemoresistance, which was associated with a selective inhibition of the HMTases SUV39H1, SUV39H2, and G9a/GLP that exhibit redundant functions in H3K9 trimethylation and *FAS* transcriptional silencing [83]. Verticillin A (**13**) sensitized metastatic human colon carcinoma cells to Fas-mediated apoptosis, exhibiting greater efficacy than decitabine and vorinostat, increasing also death receptor 5 (DR5) expression to effectively overcome resistance to DR5 agonist drozitumab-induced apoptosis [83]. The same group suggested also that **13** act as an apoptosis sensitizer, at least partially, through the activation of DNA methylation–silenced *BNIP3* transcription through a DNA demethylation–independent mechanism [83,86]. In addition to H3K9me3, verticillin A (**13**) also targets H3K4me3 in pancreatic cancer cells, as treatment of tumor-bearing mice decreased the H3K4me3 levels in the *cd*274 promoter region in the orthotopic tumor tissues [84]. Verticillin A (**13**) dramatically suppressed human PDAC growth, a sub-lethal dose effectively overcoming human PDAC cell resistance to gemcitabine [87]. Inhibition of PDAC growth upon treatment with **13** appears to act, at least partially, through the activation of the intrinsic apoptosis pathways, as it was found to interfere with the levels of the pro-apoptotic Bak, Bax and Bim and the antiapoptotic Bcl-x, Mcl-1 and FLIP regulatory genes, via downregulation of H3K4me3 and H3K9me3 levels [87].

Verticillin A (**13**) appears to cause distinct impacts on cell cycle progression, depending on the cell type being investigated, reflecting discrepancies in utilized pathways, differential metabolic processes, as well as dissimilarities in genetic and proteomic expression. While **13** was found to induce G_2_ cell cycle arrest in SW620 colon cancer cells, no changes in cell cycle progression were observed in HepG2 liver carcinoma cells [86] and in karyotypically complex soft tissue sarcoma (STS) cell lines [88]. In vitro and in vivo studies demonstrated that verticillin A (**13**) increased cleaved caspase-3 and induced a decrease in the Ki67 proliferation expression in malignant peripheral nerve sheath tumor (MPNST) cells and xenograft models, independent of cell cycle arrest [88].

Unlike **13 [79]**, 11,11’-dideoxyverticillin A (**14**) was found to act as a prominent inhibitor of tyrosine kinase activity of the epidermal growth factor receptor (EGFR) and vascular endothelial growth factor receptor-1/fms-like tyrosine kinase-1 (VEGFR-1/Flt-1) in a low nM range [89]. Exposure to **14** inhibited EGF-induced phosphorylation of EGFR, HER2 and Erk1/2 in EGFR-overexpressed MDA-MB-468 and HER2-overexpressed SK-OV-3 cells [89]. 11,11’-Dideoxyverticillin A (**14**) appears to be particularly cytotoxic against human breast tumor cells, potently inhibiting the proliferation of a panel of four cell lines with an average IC_50_ value of 0.2 µM. In fact, at low µM concentrations, a pro-apoptotic effect was noted by the accumulation of MDA-MB-468 cells in the G_2_/M phase of the cell cycle [89]. Antitumor effects were also observed in vivo, causing a significant reduction on tumor weight in mice sarcoma 180 and hepatoma 22 [89]. The same group described also that **14** is a structurally novel antiangiogenic agent. Following treatment with 11,11’-dideoxyverticillin (**14**), the secretion of VEGF from human MDA-MB-468 breast carcinoma cells was lowered, significantly suppressing VEGF-induced tyrosine phosphorylation of the endothelial cell-specific receptors Flt-1 and KDR/Flk-1 [90]. 11,11’-Dideoxyverticillin (**14**) was further able to reduce VEGF-stimulated human umbilical vein endothelial cells (HUVEC) proliferation and antagonized VEGF-mediated rescue of serum-deprived HUVECs, as well as to inhibit tube formation by HUVECs and to repress their mobility [90]. In vitro results were mirrored in vivo, as the formation of VEGF-induced rat aortic capillary sprouts was inhibited, suppression of new vessel growth into Matrigel plugs implanted in mice being observed as well [90].

The asymmetric dimer verticillin B (**16**) (Figure 4) was also identified in a marine fungal strain, being purified from an extract obtained from *Nectria inventa*, collected from a sediment obtained below 600 meters in Monterey Bay, California [91]. Exposure to **16** led to a potent trypanocidal effect (IC_50_ = 7 nM) towards the whole cell parasite *Trypanosoma brucei* [91]. Later, the group of Peter Proksch reported the isolation of verticillin D (**17**) (Figure 4) from an EtOAc extract of the endophytic fungus *Bionectria ochroleuca*, collected from leaf tissues of the mangrove plant *Sonneratia caseolaris* from Hainan island, China [92]. Verticillin D (**17**) exhibited potent in vitro cytotoxicity towards murine lymphoma L5178Y cells with an IC_50_ below 0.1 µM [92]. First described in a terrestrial strain of *Gliocladium catenulatum* along with two additional analogues [93], **17** was initially ascribed as an antibacterial agent, particularly active against wild and methicillin-resistant *Staphylococcus aureus* (MRSA) strains [93,94,95].

**Figure 5:**
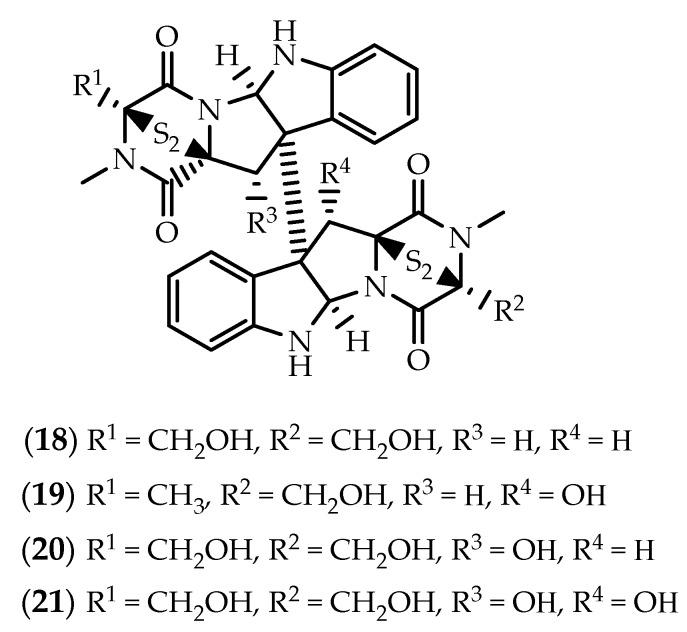
Structures of chaetocin (**18**) and melinacidins II-IV (**19**–**21**).

Structurally similar to verticillins **13**–**15**, but deriving from two molecules of L-serine instead of L-alanine, chaetocin (**18**) (Figure 5) was also obtained from the marine-derived fungus *Nectria inventa* [91]. First reported nearly 50 years ago from a strain of *Chaetomium minutum* [96,97], **18** has excited research predominantly due to its ability to inhibit SU(VAR)3-9 [98]. Imhof and coworkers screened around 3000 compounds for inhibitory activity towards recombinant *Drosophila melanogaster* SU(VAR)3-9 protein, discovering chaetocin (**18**) as the first histone lysine methyltransferase (HKMT) inhibitor, specifically inhibiting the enzymatic activities of HKMTs belonging to members of the SUV39 family, including SUV39H1, dSU(VAR)3-9, G9a, DIM-5, GLP, and ESET [98,99,100]. Readers are invited to take a gander on the review by Sodeoka and colleagues covering the chemistry and biological properties of chaetocin (**18**) up to 2012 [101]. 

Chaetocin (**18**) is mainly reputed as an epigenetic agent through the pharmacological inhibition of SUV39H1, which has been shown to be a promising therapeutic strategy for inhibiting the growth of several human cancer cells [102,103]. Inhibitory effects of **18** towards SUV39H1 were found to provoke endoplasmic reticulum (ER) stress and results in the upregulation of the activating transcription factor 3 (ATF3) and C/EBP homologous protein (CHOP) in non-small cell lung cancers, suggestive of DR5-dependent apoptosis [104]. Synergistic cytotoxicity towards acute myeloid leukemia cells has been reported when combined with histone deacetylase and BET inhibitors [103]. More relevant, combination of chaetocin (**18**) with Aurora kinase A (AURKA) inhibitors was found to be effective at inhibiting the growth of PDAC cells both in vitro and in vivo, via mitotic catastrophe characterized by aberrant mitotic checkpoint signaling and decreased centromeric H3K9 methylation [105]. While the combination of chaetocin (**18**) with autophagy inhibitors proved to be inefficient in adenocarcinomic human alveolar basal epithelial A549 cells [106], Jung et al. [107] observed that **18** was able to elicit both apoptosis and autophagy in human hepatoma cell lines, as suggested by the accumulation of LC3-II levels and increased GFP-LC3 puncta. Relevantly, suppression of autophagy enhanced caspase-dependent apoptotic cell death in hepatoma cells, which may dictate that there might be cell type specificity [107]. 

Chaetocin (**18**) appears to have a multiple role in cancer cells as it is able to induce also cellular oxidative stress and apoptosis. In fact, **18** regulates SUV39H1 activity in a ROS-dependent manner and influences the expression of death-receptor-related genes resulting in death receptor-dependent apoptosis [108]. Chaetocin (**18**) was also identified as an inhibitor of the redox enzyme thioredoxin reductase, thereby accounting for its capacity to induce cellular oxidative stress and eradicate tumor cells [106,109]. As oxidative stress is an important regulator of apoptosis and metabolic reprogramming, both pathways are affected by chaetocin (**18**) treatment. Induced caspase-dependent apoptosis via the excessive production of ROS was observed both in vitro and in vivo in myeloma [109,110,111], glioblastoma [112], ovarian [113] and intrahepatic cholangiocarcinoma cells [114]. In lieu of these findings, it has been reported that chaetocin (**18**) can also inhibit the production of ROS in a SIRT1-dependent manner in myocardial cells both in vivo and in vitro [115].

Chaetocin (**18**) may not only directly target cancer cells, but also indirectly inhibit tumor growth by reducing angiogenesis at the tumor microvasculature level. Chaetocin (**18**) has received further attention as it is able to inhibit the transactivation potential of hypoxia-inducible factor (HIF)-1α by attenuating its binding to p300, and thereby inhibiting the growth of hepatoma cells [116,117]. Data from rat aortic ring assays demonstrated that **18** led to a decrease in microvessel outgrowth at 8 nM indicating antiangiogenic properties, and co-immunoprecipitation experiments showed that these effects are due, at least in part, to disruption of the HIF-1α/p300 complex [118]. Early studies demonstrated that systemic administration of **18** disrupts the HIF pathway inhibiting the ability of tumors to adapt to hypoxia [116,117]. Downstream effects of inhibiting the HIF-1α/p300 interaction include decreased levels of secreted VEGF, and the subsequent downregulation of glycolytic genes *LDHA* and *ENO1*, suggesting that they played a role in inhibiting cell survival under hypoxia and promoting cell death in hypoxic areas [118]. 

Worth to mention the study by Vo et al. [119] suggesting that chaetocin (**18**) can be used as a source of antigens for loading into dendritic cells to enhance myeloma-specific antitumor immune responses. Dendritic cells loaded with **18** potently inhibited regulatory T cells and activated myeloma-specific cytotoxic T lymphocytes, via the upregulation of heat shock protein (HSP) 90 and the cancer testis antigens MAGE-A3 and MAGE-C1/CT7 in myeloma cells [119].

While mainly attracting the attention from cancer researchers, **18** has been shown to display a wide range of additional pharmacological properties, frequently associated with its ability to inhibit SU(VAR)3-9. Chaetocin (**18**) was found to improve the prognosis in Dahl salt-sensitive rats with heart failure [120]. It was observed that **18** delayed the transition from hypertrophy to heart failure, caused the restoration of mitochondrial function-related gene expression in failing hearts, and prolonged animal survival [120]. The use of HMT inhibitors as antitrypanosomal agents was also suggested based on the effects of **18** towards *Trypanosoma cruzi*. Chaetocin (**18**) inhibited cell proliferation and arrested cell cycle on G_2_/M phase of *T. cruzi* epimastigotes, nucleolar disassembly induced by the reduction of rRNA transcription being also described [121]. Bae et al. [122] reported that chaetocin (**18**) inhibits melanogenesis in B16F10 mouse melanoma cells via suppressing the protein level of microphthalmia-associated transcription factor (MITF) and followed by activation of the extracellular signal-regulated kinases (ERK) signaling pathway. Such results suggest **18** potential cosmeceutical utility and also as a topical agent for treatment of hyperpigmentation disorders.

The melinacidins II–IV (**19**–**21**) (Figure 5) were isolated from cultures of *Corollospora pulchella*, a marine fungus isolated from a sand sample from Japan [123], **21** (also known as 11,11’-dihydroxychaetocin) [124] being also reported in the Rhodophyta associated fungus *Westerdykella reniformis* [125].

**Figure 6:**
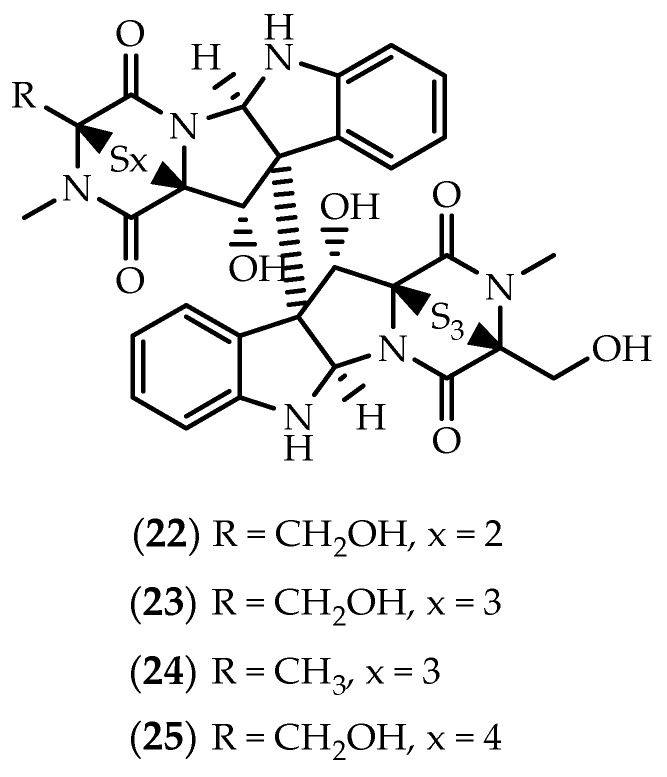
Structures of chetracins B (**22**), C (**23**), E (**24**) and F (**25**).

Despite the striking similarity with chaetocin (**18**) and melinacidin IV (**21**), and unlike the remaining epithiodiketopiperazines discussed above, chetracin B (**22**) (Figure 6) is characterized by the existence of a trisulfidge bridge assigned in the second monomeric subunit [126]. First described from an Antarctic psychrophilic fungus along with the symmetric hexasulfide homolog chetracin C (**23**) (Figure 6) [126], **22** was later isolated from the algicolous fungus *Westerdykella reniformis* obtained from Prince Edward Island, Canada [125]. In 2018, Yu and colleagues [127] identified a marine strain of the fungus *Acrostalagmus luteoalbus* (HDN13-530) as a source of chetracins C (**23**), E (**24**) and F (**25**) (Figure 6). 

Chetracins **22**–**25** were shown to be potent in vitro cytotoxic agents towards a series of cancer cell lines, in low µM or nM concentrations [126,127]. However, their pharmacological interest stems predominantly from their ability to act as HSP90 inhibitors [127,128]. As chaetocin (**18**), **22**–**25** inhibit HSP90 by binding to the C-terminal, leading to a reduction in levels and active forms of the client oncoproteins EGRF, Stat3, Akt and Erk [127,128].

**Figure 7:**
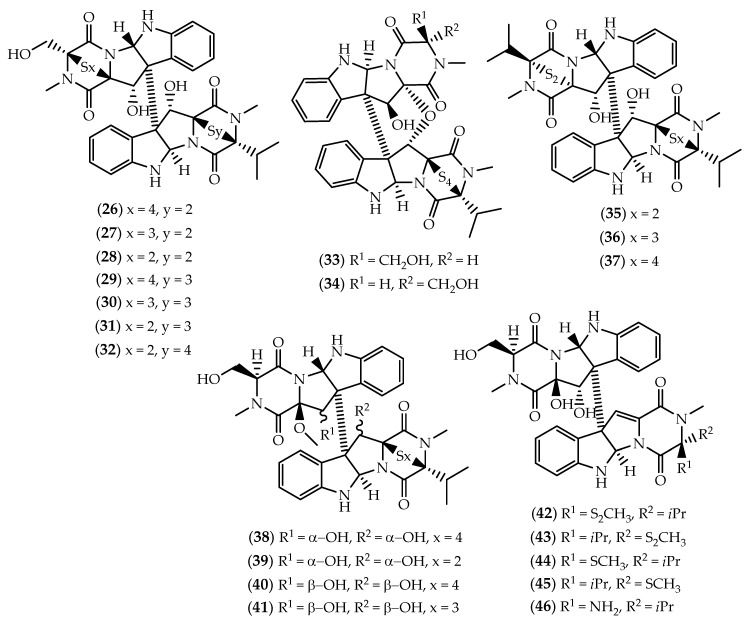
Structures of leptosins (**26**–**46**).

Between 1994 and 2005, Numata and colleagues described the cytotoxic leptosins from a strain of the fungus *Leptosphaeria* sp., isolated from the surface of the brown alga *Sargassum tortile* collected in Tanabe Bay, Japan [129,130,131,132,133,134]. So far including 21 members, leptosins correspond to the largest subset of epipolythiodiketopiperazine dimers. Leptosins exhibit a complex structural architecture, characterized at least by one valine residue, but with a varying number of sulfur atoms in the thio bridge. Based on structural similarity, dimeric leptosins can be divided into six groups. Displaying the same basic structural skeleton, leptosins A–C (**26**–**28**) [129], G (**29**), G_1_ (**30**), G_2_ (**31**) and H (**32**) [131] (Figure 7) solely differ in the number of sulfurs contained in the polythio bridges. With a reduced degree of freedom, the epimers leptosin I (**33**) and J (**34**) (Figure 7) display a C12–C11’ ether linkage [130]. Leptosins K (**35**), K_1_ (**36**) and K_2_ (**37**) (Figure 7) differ in the stereochemistry of one polythio bridge and both monomeric units contain valine residues [132]. Displaying the cyclic sulfide functionality only in one monomeric unit, leptosins M (**38**), M_1_ (**39**), N (**40**) and N_1_ (**41**) (Figure 7) are included in another group [133]. The most recently reported leptosins O–R (**42**–**45**) (Figure 7) lack the sulfide bridge, while leptosin S (**46**) (Figure 7) stands alone as the sole sulfur-deficient member of the subset [134].

Dimeric leptosins are generally toxic to lymphocytic leukemia P388 cells, most of them proving to be more efficient than mitomycin C [129,130,131,132,133,134]. Additional experiments revealed that leptosins A (**26**) and C (**28**) exhibited low nM potency towards the human pancreatic MIA PaCa-2 cancer cell line [135], both being also able to suppress tumor growth in mice bearing sarcoma 180 ascites [129]. Additionally, **28** was found to induce apoptosis through the inhibition of topoisomerase I and the Akt/protein kinase B survival pathway in human lymphoblastoid RPMI8402 and embryonic kidney cells [136]. Apart from the potent cytotoxic effects upon P388 cells, leptosin M (**38**) proved to exert relevant cytotoxicity towards 39 human cancer cell lines from the HCC panel of the Japanese Foundation for Cancer Research, and specifically inhibited topoisomerase II and the protein kinases PTK and CAMKIII [133]. The pattern of differential cytotoxicity was evaluated using the COMPARE program, suggesting that the mode of action for leptosin M (**38**) might be different from conventional chemotherapeutic drugs [133].

### 2.2. Dimers Joined Through Distinct Linkages

**Figure 8:**
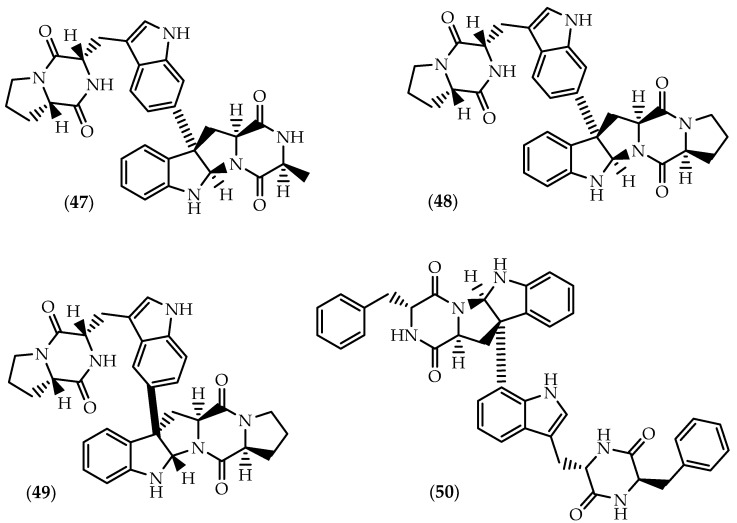
Structures of naseseazines A-C (**47**–**49**) and asperazine (**50**).

The Fijan marine sediment-derived actinomycete *Streptomyces* sp. CMB-MQ030 was the source of naseseazines A (**47**) and B (**48**) (Figure 8) [137], their stereochemical revision being later attained by Kim and Movassaghi [138]. In 2016, Buebenbender et al. [139] isolated naseseazine C (**49**) (Figure 8) along with **47** and **48** from an EtOAc extract of the *Streptomyces* sp. strain USC-636 collected from a marine sediment obtained on the Sunshine Coast, Australia. Carroll’s group further reported that the first proposed structure for **49** was wrongly assigned as the C3/C7’ isomer, concluding that *iso*-naseseazine B [140] corresponds to naseseazine C (**49**). Naseseazines are characterized by distinct heterodimeric frameworks in which the indole units bind via C7’ (**47** and **48**) or C6’(**49**) to the pyrroloindoline monomer. Unlike **48** and **49**, in naseseazine A (**47**) one L-proline is replaced by L-alanine in the pyrroloindoline unit.

The antiplasmodial activity of naseseazines A-C (**47**–**49**) was assayed against a chloroquine-sensitive strain of *Plasmodium falciparum*, but solely **49** proved to be moderately active suggesting that the C3-C6’ linkage and/or the change in regiochemistry in subunit A could be responsible for the enhanced bioactivity [139]. Naseseazine C (**49**) was shown to display also weak antifungal activity against fluconazole-resistant *Candida albicans* [140]. The biosynthesis of naseseazine C (**49**) was recently linked to the CDPS-containing cluster *nasc*A-*nasc*B, the cytochrome P450 NascB being responsible for the dimerization through a biradical mechanism for the C3-aryl bond formation with both regio- and stereospecificity [141].

Characterized by a unique C3-C8’ juncture, asperazine (**50**) (Figure 8) is another member of the rare group of dimeric diketopiperazines featuring a linkage between C3 of subunit A and the tryptophan aromatic ring of subunit B. The heterodimer was isolated in minute quantities from cultures of *Aspergillus niger* (#94-1212) isolated from the sponge *Hyrtios proteus* collected in the Dry Tortugas National Park in Florida [142,143]. Asperazine (**50**) displayed modest cytotoxicity against liver hepatocellular carcinoma HepG2 and cervical carcinoma CaSki cells [144], but unlike the original isolate [142], synthetic asperazine was not shown to exert significant antileukemic activity in the Corbett-Valeriote soft agar disk diffusion assay [145]. Worth to mention the inhibitory effects on HIV-1 replication in C8166 cells following the treatment with **50**, displaying stronger antiviral effect than indinavir [146]. Further studies revealed antifungal activities against the phytopathogens *Fusarium oxysporum* f.sp. *lycopersici* [147], *Botrytis cinerea*, *Gibberella saubinetti*, *Magnaporthe grisea* and *Alternaria solani* [148].

**Figure 9:**
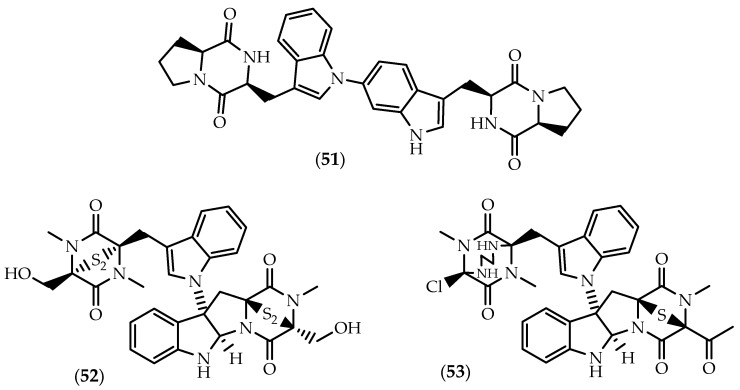
Structures of aspergilazine A (**51**), chetomin (**52**) and cristazine (**53**).

Aspergilazine A (**51**) (Figure 9), a bis-indole derivative with a rare C6-N1′ linkage, was reported by Cai et al. [149] from the mangrove root soil *Aspergillus taichungensis* ZHN-7-07. A weak antiviral effect against influenza A (H_1_N_1_) virus was reported in the same study [149].

Widely reported from *Chaetomium* spp., the well-known cytotoxic agent chetomin (**52**) (Figure 9) was also found to occur in marine-derived strains, namely in *Chaetomium cristatum* isolated from the sediments of marine mudflat collected at Suncheon Bay, Korea [150]. Originally reported in the 1944 by Waksman and colleagues as a mixture of antibiotics named chaetomin [151,152], its planar structure bearing a C3-N1′ bond was unknown until the late 1970s [153,154,155,156], being only fully elucidated by Kikuchi et al. [157] in 1982. Upon request of the Editor of Chemical Abstracts, chaetomin was later renamed chetomin (**52**) [153].

Chetomin (**52**) was reported as a mycotoxin implicated in ovine ill-thrift in Nova Scotia, Canada [153,158] due to its potent antibacterial properties towards rumen flora [152,159,160,161]. However, **52** became a pharmacologically relevant metabolite as a well-characterized and selective inhibitor of HIF-1α transcriptional activity. It targets the transcriptional co-activator p300 by ejecting the zinc ion from its CH1 domain, disrupting the interactions with the C-terminal transactivation domain of HIF-1α [116,162]. Consequently, chetomin mitigates hypoxia-inducible transcription of downstream signaling moieties [163,164], biological implications including antiangiogenic and antitumor effects.

While relatively less effective than chaetocin (**18**), chetomin (**52**) also exhibited significant antiangiogenic properties derived from the disruption of the HIF-1α/p300 complex [116,118]. Chetomin (**52**) exhibited antitumor activity in human myeloma cell lines and primary multiple myeloma cells from patients, suggestive of potential clinical value in multiple myeloma patients characterized by a high EP300 and HIF-1α expression [165]. Inhibition of HIF-1α by **52** effectively reduces CA9 and VEGF mRNA expression [166], enhancing the radiation response under severely hypoxic conditions in HT 1080 human fibrosarcoma cells and U251MG and U343MG glioma cells [163,166].

Due to the inhibition of HIF-1α, chetomin was (**52**) reported to decrease invasiveness in MDA-MB-231 triple negative breast cancer cells under hypoxic conditions [167]. More recently, **52** was also found to induce apoptosis in human triple-negative breast cancer cells by mitochondrial dysfunction, through the inhibition of PI3K/mTOR induced ER stress and promotion of calcium overload [168]. In fact, additional molecular mechanisms underlying chetomin (**52**) anticancer effects have been reported. Anticancer effects of **52** were suggested to derive from the specific activation of mutant p53^R175H^, restoring wildtype p53 transactivation and upregulating MDM2, p21 and PUMA expression [169]. Chetomin (**52**) selectively inhibited the growth of tumor cells harboring p53^R175H^ but not p53^R273H^ in mouse xenograft models [169]. In another study it was observed that co-treatment with tumor necrosis factor-related apoptosis-inducing ligand (TRAIL) synergistically induced apoptosis in urogenital PC-3, Caki-1 and UM-UC-3 cancer cells, inducing the activation of caspase-3, -8, -9 and -10 [170]. Yano et al. [170] suggested that the TRAIL-induced apoptosis occurred via downregulation of the X-linked inhibitor of apoptosis (XIAP) in a proteasome-dependent manner. Chetomin (**52**) was also described as a potent inhibitor of H3K9 methyltransferases, displaying stronger HMTase inhibitory activity than 11,11’-dideoxyverticillin A (**14**), against G9a and Suv39h1H320R [171].

Enhancement of the antiviral response was also found to be dependent on HIF, as chetomin (**52**) increased the sensitivity of renal carcinoma 786-O cells to vesicular stomatitis virus (VSV)-mediated cytolysis [172]. Immunosuppressive activities were also reported against Con A-induced (T-cells) and LPS-induced (B-cells) proliferations of mouse splenic lymphocytes, following chetomin (**52**) treatment at low µM concentrations [173]. Chetomin (**52**) was identified as the first naturally-occurring antagonist of the C-C chemokine receptor type 2 (CCR2), known for the involvement in inflammatory processes and infectious diseases. It was found to selectively inhibit the binding of MCP-1 to CCR2 (CHO membrane) using human monocyte cells harvested from Leukopacks [174].

While the biosynthetic origin of the C3-N1’ linkage is not well understood, Welch and Williams [175] proposed a route to the biosynthesis of chetomin (**52**) through the convergent and enantioselective synthesis of an intermediate. 

Cristazine (**53**) (Figure 9) bears the same C3 to N1’ bond as chetomin (**52**), but the disulfide bridge is replaced by a monosulfide bridge, differing also on the other monomeric half characterized by the 1,2-ethanediamino bridged diketopiperazine moiety [150]. Cristazine (**53**) was purified along with **52** from an extract of the marine-sediment-derived *Chaetomium cristatum* [150]. Low µM cytotoxicity was observed for human cervical carcinoma HeLa cells [150], further experiments from the same groups evidencing that **53** triggered apoptotic cell death via the Type I death receptor pathway in human epidermoid carcinoma A431 cells [176]. Cristazine (**53**) induced the activation of caspase-3, -6, -7, and -8 and the subsequent cleavage of FLIP, RIP, PARP, DFF, and lamin A, causing also cell cycle arrest in the G_1_/S phase and the upregulation of the inhibitory proteins of cyclin-dependent kinases [176]. 

**Figure 10:**
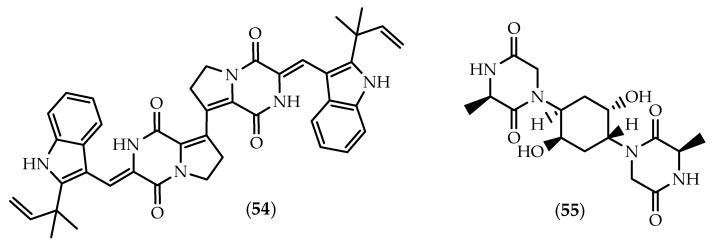
Structures of brevianamides S (**54**) and (**55)**.

The dimeric brevianamide S (**54**) (Figure 10) and a series of monomeric brevianamides were sourced from a strain of *Aspergillus versicolor* isolated from a sediment collected from the Bohai Sea, China [177]. Scientific soundness derived not only from its dimeric C8-C8’ linkage, but also due to the selective activity against the Bacille Calmette-Guérin (BCG) strain of *Mycobacterium bovis*, which suggested a new antitubercular mechanism of the action [177].

Isolated from the echinoderm *Pentaceraster regulus* obtained in the Indian Ocean, **55** (Figure 10) is the only dimeric diketopiperazine being reported from a marine macroorganism to date [178].

## 3. Discussion

### 3.1. Considerations on the Occurrence of Marine Diketopiperazine Dimers

The wide set of putative modification enzymes found within the NRPS and CDPS gene clusters, allowing the formation of dimeric structures and other structural modifications, evidences that the synthesis of highly modified diketopiperazines seems to be the norm rather the exception [179]. As previously mentioned, the diketopiperazine motif is normally assembled by NRPSs in fungi, whereas in bacteria mainly by tRNA-dependent CDPSs. However, and as corroborated by the current review, their structural and functional differences seem to be related with the higher abundance and structural diversity of diketopiperazines obtained from fungal sources [26]. Mainly characterized in bacterial genomes, CDPSs hijack aa-tRNAs, thus not requiring the activation of the amino acids, and are therefore limited to the 20 canonical amino acids charged on tRNAs [35]. In contrast, the range of amino acids that can be incorporated by NRPSs is much wider, as they also use distinct building blocks, including non-proteinogenic amino acids, contributing to the wide structural diversity of monomeric and dimeric diketopiperazines obtained from fungi [180,181]. Additionally, in CDPS biosynthetic routes, chemical modifications can only be introduced after diketopiperazine formation, while in NRPS pathways, substrates can be modified on the enzyme by accessory domains leading to a wider structural complexity [26,35]. 

The discovery that a significant number of diketopiperazines that were previously described as being sourced from fungi can be also produced by CDPSs in bacterial strains evidences that the sourcing organism of some of these metabolites can frequently be difficult to ascribe, and that taxonomically distant species can produce similar diketopiperazine dimers [34,182]. For example, and as mentioned by Schenke et al. [183], the isolation of verticillin A (**13**) from cultures of a strain of *Gliocladium roseum*, a mycoparasite of *Verticillium*, evidenced that the original fungal source might have been confused with another fungus or contaminated with a *Gliocladium* colonist. It is also interesting to observe that while originally reported from a marine strain of an *Aspergillus* sp. [149], aspergilazine A (**51**) was later isolated from the marine *Streptomyces* sp. SMA-1 [140]. Also, Urbatzka’s group suggested that the epitetrathiodiketopiperazine monomer leptosin F can be produced by cyanobacterial strains [184], in contrast to previous reports indicating that leptosins were exclusively sourced by fungi [129,130,131,132,133,134,135,185]. 

Although marine-derived microorganisms have the capability of generating novel secondary metabolites, many of those compounds and their analogues are also produced by terrestrial strains [186,187]. It is worth highlighting that from the 55 dimeric diketopiperazines described here as occurring in marine sources, 20 have been also reported as being produced by terrestrial microorganisms. In fact, several studies suggest than even marine obligate species derived from terrestrial ancestors [188]. However, the biosynthetic machinery of microorganisms is highly dependent on marine influences on the formation of structurally distinct metabolites in comparison with terrestrial strains [189,190]. The recognition that carbon and nitrogen sources, light, temperature, and pH greatly affect metabolite output is well illustrated by the wide range of conditions used to elicit metabolite production in industrial settings [190]. As recently mentioned by Amend et al. [191], environmental DNA-based surveys in marine habitats has unveiled inconspicuous microbial diversity from animal hosts and ocean sediments, thus shedding light on chemical dark matter. As such, one should expect that the pronounced progress in microbial genome sequencing and metagenomics, as well as the subsequent identification of previously uncharacterized NRPSs and CDPSs, will certainly lead to an exponential increase on the discovery of new dimeric diketopiperazines from marine sources [192,193,194].

### 3.2. Pharmacological Highlights and the Importance of Being Dimeric

Complex structural modifications driven by NRPSs and CDPSs markedly influence the biological effects of the respective dimeric diketopiperazines through the alteration of the hydrophobicity, shape, or rigidity of the scaffold, which is indicative of varied functions in the producing organisms [37,179]. Despite the exponential increase in the number of bioactive diketopiperazines being characterized from natural sources, in particular from the marine environment, relatively little is known about their ecological functions [195]. Suggested roles include their involvement in cell-to-cell communication phenomena such as quorum-sensing in bacteria [196,197,198], but also interspecies and transkingdom signaling [199,200,201].

In fact, so far, little is known by researchers about why dimeric diketopiperazines come into being. However, as clearly noted in this review, many of these structurally elegant metabolites possess a “*Janus-faced*” range of biological effects, not only toxic, but also frequently with relevant pharmacological properties and subsequent therapeutic utility. 

The clinical utility of several dimeric epithiodiketopiperazines has been hampered due to the general toxicity associated with the sulfide moiety and associated oxidative burst, but recent studies may change the paradigm. In fact, cytotoxicity of several epithiodiketopiperazines has not been always accompanied by elevated cellular ROS levels, evidencing a certain degree of selectivity [202]. Their potent cytotoxicity towards cells with elevated levels of glutathione affects their application to overcome chemoresistance caused by elevated levels of glutathione and their mitochondrial detoxifying enzymes [72,203]. Furthermore, long-term cytotoxicity of epithiodiketopiperazines appears to be greatly reduced by replacing the hydroxymethyl group with a methyl group [204]. In a SAR study on chaetocin (**18**) derivatives, Sodeoka’s group also demonstrated that its simple derivatives were significantly less toxic, but were also effective inhibitors of G9a [205]. Nevertheless, the discovery of dimeric epidithiodiketopiperazines vastly contributed to understanding the involvement of their biological targets in the development of certain types of cancer, enabling new chemotherapeutic approaches.

As previously mentioned, verticillin A (**13**) targets six HMTases indicating that it is potentially toxic at high doses in vivo [86], but an extreme increase in human PDAC cell sensitivity to gemcitabine-induced growth suppression was observed following the treatment with sub-lethal doses [87]. In fact, verticillin A (**13**) appears to be an effective epigenetic agent for targeting 5-FU resistance in human patients with metastatic colorectal cancer and gemcitabine resistance in PDAC patients, and thus holds great promise for further development as an “epi-drug” candidate [83,87]. Nevertheless, while clinical testing is still necessary to demonstrate the specificity and toxicity of verticillin A (**13**), relevant findings concerning the evasion and progression of colon carcinoma and PDAC were disclosed. Verticillin A (**13**) was found to be a new selective HMTase inhibitor that inhibits H3K9me3 to restore Fas expression, indicating that H3K9me3-mediated *FAS* transcription silencing is a dominant mechanism by which colon cancer cells evade host cancer immune surveillance [83]. Furthermore, **13** downregulated H3K4me3 and H3K9me levels in PDAC cells, determining that a panel of apoptosis regulators was deregulated by epigenetic mechanisms leading to an apoptosis-resistant phenotype in PDAC cells, suggesting that HMTase targeting may be an effective approach to overcome their resistance to chemotherapy [87].

While the discovery of chaetocin (**18**) as the first selective inhibitor of the SUV39H family is relevant per se, it is worth highlighting two main findings associated with **18**. The new combinatorial therapy based on the AURKA inhibitor MLN8237 and chaetocin (**18**) provided significant mechanistic value as it relates to the development of new therapies based on the combined targeting of a genetic-to-epigenetic pathway via a cytotoxic mechanism that involves perturbation of normal mitotic progression to end in mitotic catastrophe [105]. Chaetocin (**18**) also set the cornerstone for the potential development of novel therapies for chronic heart failure based on the inhibition of histone H3K9 methyltransferase, allowing to maintain the appropriate chromatin structure and reversing excessive heterochromatinization at repeats in the introns of critical genes for pumping function [120,206]. In fact, **18** disclosed the previously unrecognized role for SUV39H linking SIRT1 trans-repression of myocardial infarction [115].

Chetomin (**52**) has been also playing a tremendous role on the elucidation of the mechanisms underlying the invasiveness of specific types of cancer cells, namely the preponderant role of hypoxia in ovarian [164] and triple-negative breast [167] ovarian cancer.

The wide range of pharmacological effects herein reviewed is not surprising, as diketopiperazines frequently mimic preferential peptide conformations, with the two hydrogen-bond donor and acceptor sites further favoring interactions with a wide set of receptors [207,208]. In fact, diketopiperazines have attracted much interest in pharmaceutical development due to the three-dimensionality, and the possibility to introduce several substituents to the core ring as well as to the respective side chains of the constituent amino acids, constituting a clear advantage over conventional molecules developed through combinatorial chemistry [207]. Furthermore, the rigid six-membered ring confers conformational rigidity and stability at low pH, and is associated with low vulnerability to enzymatic degradation, further increasing their ability to specifically interact with biological targets [208,209].

Dimeric diketopiperazines are privileged structures as they encompass a diketopiperazine scaffold, their pharmacological potency being also frequently boosted in comparison with the corresponding monomeric counterparts. As it was found that the symmetry of WIN 64821 (**2**) was not a structural requisite for high NK-1 binding affinity, a series of synthetic monomers were synthesized for SAR studies [53]. While the binding mode of **2** was not fully elucidated, simplified synthetic derivatives were found to be inactive or to display 100-fold less binding affinity towards NK-1 receptor, evidencing that solely one-half of the symmetric dimer is not sufficient for substance P antagonism [50,53]. Also, the monomeric leptosins were found to be drastically less cytotoxic than dimeric members (**26**–**28**) towards cultured P388 lymphocytic leukemia cells [129]. A series of structurally simplified analogues were synthesized in order to identify the structural requirements of chaetocin (**18**) for G9a and thioredoxin reductase inhibitory activity [205]. SAR results highlighted the importance of the disulfide functionalities in **18**, and while monomeric derivatives seem to be also available as pharmacophores for G9a inhibition, almost no inhibition upon thioredoxin reductase has been observed, in contrast with the dimeric parent compound. Furthermore, simplified monomers were also unable to rival **18** cytotoxicity upon human leukemia HL-60 cells [205]. In fact, dimeric compounds very often render better “drug-like” properties as a result of their potential to bind two distinct individual binding sites on a single receptor or a defined site on two separate monomers of a dimeric protein [210]. Furthermore, the dual interaction of a dimeric agent can produce also enhanced selectivity when properly crafted, constraining the molecule in an optimal orientation for binding the second ligand [210]. As seen with dimeric diketopiperazines, several studies on naturally-occurring or synthetic dimers evidenced not only increased potency, but also high-affinity interactions and additional complementary actions [211,212,213,214,215,216].

Despite the relevant pharmacological properties and potentially improved pharmacokinetic parameters, supply of marine dimeric diketopiperazines has proven to be challenging. The large supply of metabolites from marine sources frequently demands convenient approaches that enable the provision of the necessary quantities of material to complete in vitro studies and initiate preclinical evaluation [217]. The total synthesis of (+)-11,11’-dideoxyverticillin A (**14**) was achieved by Movassaghi’s group based on Kirby’s biosynthetic hypothesis [218] and provided the foundation stone for the chemical synthesis of dimeric epithiodiketopiperazines but also sulfur-deficient dimers [66,219,220,221,222]. The synthesis of dimeric diketopiperazines has been successfully achieved by several groups, often resulting in the revision of their structures [62,68,138]. Such synthetic routes have been translated in the development of new strategies to address strenuous structural features such as C3/C3’ vicinal quaternary centers, C3-Csp^2^ bonds, heterodimeric linkages and the incorporation of the cyclic sulfide moiety [223]. Additionally, these approaches allow a convenient supply for pre-clinical and clinical development, enabling the development of unnatural derivatives with optimized pharmacological properties [204,224,225,226,227]. A second important aspect concerning the efficient production of dimeric diketopiperazines deals with the elucidation of the ditryptophenaline (**1**) [48] and naseseazine C (**49**) pathways [141]. As bacterial fermentation is often more efficient and cost-effective than chemical routes, their discovery paves the way for the biosynthetic assembly of novel dimeric diketopiperazines in large amounts through heterologous expression in engineered biocatalysis systems [32,192].

## 4. Conclusions

Dimeric diketopiperazines encompass a vast spectrum of biological properties pointing to various therapeutic possibilities, with additional chemical characteristics making them attractive scaffolds for drug development. So far, little has been learnt on how the intriguing dimeric connectivity is fashioned, with biosynthetic machineries being also able to produce distinguishable structural arrangements, allowing natural products to be assembled and tailored in a step-wise fashion, as demonstrated by the peculiar and elegant structure of diketopiperazine dimers.

The dimeric subset of diketopiperazines has greatly increased in the past 10 years, particularly due to their isolation from marine-derived microorganisms, and it is likely that hundreds of additional variants exist in seas. The tremendous progress in genome mining and computational discovery approaches focusing on the marine environment will certainly unveil some of these variants in the upcoming years, with an even more intriguing structural complexity as well as with biological properties that may have their medicinal chemistry developed from leads to new clinical drugs.

With the current review, we hope to fuel further studies on the bioprospection of marine organisms as producers of structurally intriguing metabolites as dimeric diketopiperazines, but also to motivate marine natural product chemists to bring back some of these precious metabolites from the shelf to the laboratory bench in order to fully assess their pharmacological properties and potential therapeutic utility.

## References

[1] Carroll A.R., Copp B.R., Davis R.A., Keyzers R.A., Prinsep M.R. (2019). Marine natural products. Nat. Prod. Rep..

[2] Pereira R.B., Evdokimov N.M., Lefranc F., Valentão P., Kornienko A., Pereira D.M., Andrade P.B., Gomes N.G.M. (2019). Marine-derived anticancer agents: Clinical benefits, innovative mechanisms, and new targets. Mar. Drugs.

[3] Pereira R.B., Dasari R., Lefranc F., Kornienko A., Kiss R., Gomes N.G.M., Andrade P.B., Valentão P., Pereira D.M. (2017). Targeting enzymatic pathways with marine-derived clinical agents. Natural Products Targeting Clinically Relevant Enzymes.

[4] Huang R.M., Yi X.X., Zhou Y., Su X., Peng Y., Gao C.H. (2014). An update on 2,5 diketopiperazines from marine organisms. Mar. Drugs.

[5] Huang R., Zhou X., Xu T., Yang X., Liu Y. (2010). Diketopiperazines from marine organisms. Chem. Biodivers..

[6] Sano S., Nakao M. (2015). Chemistry of 2,5-diketopiperazine and its bis-lactim ether: A brief review. Heterocycles.

[7] Borthwick A.D. (2012). 2,5-Diketopiperazines: Synthesis, reactions, medicinal chemistry, and bioactive natural products. Chem. Rev..

[8] Ma Y.M., Liang X.A., Kong Y., Jia B. (2016). Structural diversity and biological activities of indole diketopiperazine alkaloids from fungi. J. Agric. Food Chem..

[9] Ressureição A.S.M., Delatouche R., Gennari C., Piarulli U. (2011). Bifunctional 2,5-diketopiperazines as rigid three-dimensional scaffolds in receptors and peptidomimetics. Eur. J. Org. Chem..

[10] Wang Y., Wang P., Ma H., Zhu W. (2013). Developments around the bioactive diketopiperazines: A patent review. Expert Opin. Ther. Pat..

[11] Sung B.J., Hwang K.Y., Jeon Y.H., Lee J.I., Heo Y.S., Kim J.H., Moon J., Yoon J.M., Hyun Y.L., Kim E. (2003). Structure of the catalytic domain of human phosphodiesterase 5 with bound drug molecules. Nature.

[12] Borthwick A.D. (2010). Oral oxytocin antagonists. J. Med. Chem..

[13] Gomes N.G.M., Lefranc F., Kijjoa A., Kiss R. (2015). Can some marine-derived fungal metabolites become actual anticancer agents?. Mar. Drugs.

[14] Hayashi Y., Yamazaki-Nakamura Y., Yakushiji F. (2013). Medicinal chemistry and chemical biology of diketopiperazine-type antimicrotubule and vascular-disrupting agents. Chem. Pharm. Bull..

[15] Mohanlal R.W., Lloyd K., Huang L. (2018). Plinabulin, a novel small molecule clinical stage IO agent with anti-cancer activity, to prevent chemo–induced neutropenia and immune related AEs. J. Clin. Oncol..

[16] Cornacchia C., Cacciatore I., Baldassarre L., Mollica A., Feliciani F., Pinnen F. (2012). 2,5 Diketopiperazines as neuroprotective agents. Mini Rev. Med. Chem..

[17] Teixidó M., Zurita E., Malakoutikhah M., Tarragó T., Giralt E. (2007). Diketopiperazines as a tool for the study of transport across the blood-brain barrier (BBB) and their potential use as BBB-Shuttles. J. Am. Chem. Soc..

[18] Kogut E.F., Thoen J.C., Lipton M.A. (1998). Examination and enhancement of enantioselective autoinduction in cyanohydrin formation by *cyclo*[(*R*)-His-(*R*)-Phe]. J. Org. Chem..

[19] Oku J.I., Inoue S. (1981). Asymmetric cyanohydrin synthesis catalysed by a synthetic cyclic dipeptide. J. Chem. Soc. Chem. Commun..

[20] Martins M.B., Carvalho I. (2007). Diketopiperazines: Biological activity and synthesis. Tetrahedron.

[21] Jia B., Ma Y., Chen D., Chen P., Hu Y. (2018). Studies on structure and biological activity of indole diketopiperazine alkaloids. Prog. Chem..

[22] Li S.M. (2010). Prenylated indole derivatives from fungi: Structure diversity, biological activities, biosynthesis and chemoenzymatic synthesis. Nat. Prod. Rep..

[23] Ruiz-Sanchis P., Savina S.A., Albericio F., Álvarez M. (2011). Structure, bioactivity and synthesis of natural products with hexahydropyrrolo [2,3-*b*] indole. Chem. Eur. J..

[24] Wang X., Li Y., Zhang X., Lai D., Zhou L. (2017). Structural diversity and biological activities of the cyclodipeptides from fungi. Molecules.

[25] Li S.M. (2009). Applications of dimethylallyltryptophan synthases and other indole prenyltransferases for structural modification of natural products. Appl. Microbiol. Biotechnol..

[26] Belin P., Moutiez M., Lautru S., Seguin J., Pernodet J.L., Gondry M. (2012). The nonribosomal synthesis of diketopiperazines in tRNA-dependent cyclodipeptide synthase pathways. Nat. Prod. Rep..

[27] Giessen T.W., Marahiel M.A. (2012). Ribosome-independent biosynthesis of biologically active peptides: Application of synthetic biology to generate structural diversity. FEBS Lett..

[28] Payne J.A.E., Schoppet M., Hansen M.H., Cryle M.J. (2017). Diversity of nature’s assembly lines—Recent discoveries in non-ribosomal peptide synthesis. Mol. BioSyst..

[29] Gondry M., Sauguet L., Belin P., Thai R., Amouroux R., Tellier C., Tuphile K., Jacquet M., Braud S., Courçon M. (2009). Cyclodipeptide synthases are a family of tRNA-dependent peptide bond–forming enzymes. Nat. Chem. Biol..

[30] Liu J., Yu H., Li S.M. (2018). Expanding tryptophan-containing cyclodipeptide synthase spectrum by identification of nine members from *Streptomyces* strains. Appl. Microbiol. Biotechnol..

[31] Sauguet L., Moutiez M., Li Y., Belin P., Seguin J., Le Du M.H., Thai R., Masson C., Fonvielle M., Pernodet J.L. (2011). Cyclodipeptide synthases, a family of class-I aminoacyl-tRNAsynthetase-like enzymes involved in non-ribosomal peptide synthesis. Nucleic Acids Res..

[32] Borgman P., Lopez R.D., Lane A.L. (2019). The expanding spectrum of diketopiperazine natural product biosynthetic pathways containing cyclodipeptide synthases. Org. Biomol. Chem..

[33] Moutiez M., Schmitt E., Seguin J., Thai R., Favry E., Belin P., Mechulam Y., Gondry M. (2014). Unravelling the mechanism of non-ribosomal peptide synthesis by cyclodipeptide synthases. Nat. Commun..

[34] Gondry M., Jacques I.B., Thai R., Babin M., Canu N., Seguin J., Belin P., Pernodet J.L., Moutiez M. (2018). A comprehensive overview of the cyclodipeptide synthase family enriched with the characterization of 32 new enzymes. Front. Microbiol..

[35] Mishra A.K., Choi J., Choi S.J., Baek K.H. (2017). Cyclodipeptides: An overview of their biosynthesis and biological activity. Molecules.

[36] Kishimoto S., Sato M., Tsunematsu Y., Watanabe K. (2016). Evaluation of biosynthetic pathway and engineered biosynthesis of alkaloids. Molecules.

[37] Giessen T.W., von Tesmar A.M., Marahiel M.A. (2013). A tRNA-Dependent two-enzyme pathway for the generation of singly and doubly methylated ditryptophan 2,5-diketopiperazines. Biochemistry.

[38] Ortiz A., Sansinenea E. (2017). Cyclic dipeptides: Secondary metabolites isolated from different microorganisms with diverse biological activities. Curr. Med. Chem..

[39] Prasad C. (1995). Bioactive cyclic dipeptides. Peptides.

[40] Gomes N.G.M., Pereira D.M., Valentão P., Andrade P.B. (2018). Hybrid MS/NMR methods on the prioritization of natural products: Applications in drug discovery. J. Pharm. Biomed. Anal..

[41] Bérubé G. (2006). Natural and synthetic biologically active dimeric molecules: Anticancer agents, anti-HIV agents, steroid derivatives and opioid antagonists. Curr. Med. Chem..

[42] Springer J.P., Büchi G., Kobbe B., Demain A.L., Clardy J. (1977). The structure of ditryptophenaline—A new metabolite of *Aspergillus flavus*. Tetrahedron Lett..

[43] Lin A.Q., Du L., Fang Y.C., Wang F.Z., Zhu T.J., Gu Q.Q., Zhu W.M. (2009). *iso*-*α*-Cyclopiazonic acid, a new natural product isolated from the marine-derived fungus *Aspergillus flavus* C-F-_3_. Chem. Nat. Compd..

[44] Sun K., Li Y., Guo L., Wang Y., Liu P., Zhu W. (2014). Indole diterpenoids and isocoumarin from the fungus, *Aspergillus flavus*, isolated from the prawn, *Penaeus vannamei*. Mar. Drugs.

[45] Zhang J., Peng J., Liu T., Xin Z. (2016). Isolation and identification of endophyte and its secondary metabolites from *Salicornia bigelovii* Torr. based on type I polyketide synthase (*PKS* I) gene. Shipin Kexue (Food Sci.).

[46] Nakagawa M., Sugumi H., Kodato S., Hino T. (1981). Oxidative dimerization of Nb-acyltryptophans total synthesis and absolute configuration of ditryptophenaline. Tetrahedron Lett..

[47] Maes C.M., Potgieter M., Steyn P.S. (1986). N.m.r. assignments, conformation, and absolute configuration of ditryptophenaline and model dioxopiperazines. J. Chem. Soc. Perkin Trans. 1.

[48] Saruwatari T., Yagishita F., Mino T., Noguchi H., Hotta K., Watanabe K. (2014). Cytochrome P450 as dimerization catalyst in diketopiperazine alkaloid biosynthesis. ChemBioChem.

[49] Barrow C.J., Sedlock S.M. (1994). 1’-(2-Phenyl-ethylene)-ditryptophenaline, a new dimeric diketopiperazine from *Aspergillus flavus*. J. Nat. Prod..

[50] Barrow C.J., Cai P., Snyder J.K., Sedlock D.M., Sun H.H., Cooper R. (1993). WIN 64821, a new competitive antagonist to substance P, isolated from an *Aspergillus* species: Structure determination and solution conformation. J. Org. Chem..

[51] Sedlock D.M., Barrow C.J., Brownell J.E., Hong A., Gillum A.M., Houck D.R. (1994). WIN 64821, a novel neurokinin antagonist produced by an *Aspergillus* sp. I. Fermentation and isolation. J. Antibiot..

[52] Ding L., Li F.C., Qin M., Qin S., Kelter G., Fiebig H.H., Laatsch H. (2008). Anti-tumor compounds isolated from marine *Aspergillus* sp.. Chin. J. Nat. Med..

[53] Barrow C.J., Musza L.L., Cooper R. (1995). Structure-activity studies of the natural product substance P antagonist WIN 64821. Bioorg. Med. Chem. Lett..

[54] Popp J.L., Musza L.L., Barrow C.J., Rudewicz P.J., Houck D.R. (1994). WIN 64821, a novel neurokinin antagonist produced by an *Aspergillus* sp. III. Biosynthetic analogs. J. Antibiot..

[55] Oleynek J.J., Sedlock D.M., Barrow C.J., Appell K.C., Casiano F., Haycock D., Ward S.J., Kaplita P., Gillum A.M. (1994). WIN 64821, a novel neurokinin antagonist produced by an *Aspergillus* sp. II. Biological activity. J. Antibiot..

[56] Overman L.E., Paone D.V. (2001). Enantioselective total syntheses of ditryptophenaline and *ent*-WIN 64821. J. Am. Chem. Soc..

[57] Xu J., Hu Q., Ding W., Wang P., Di Y. (2018). New asymmetrical bispyrrolidinoindoline diketopiperazines from the marine fungus *Aspergillus* sp. DX4H. Nat. Prod. Res..

[58] Gu B.B., Gui Y.H., Liu L., Su Z.Y., Jiao W.H., Li L., Sun F., Wang S.P., Yang F., Lin H.W. (2019). A new asymmetric diketopiperazine dimer from the sponge-associated fungus *Aspergillus versicolor* 16F-11. Magn. Reson. Chem..

[59] Cho K.H., Sohn J.H., Oh H. (2018). Isolation and structure determination of a new diketopiperazine dimer from marine-derived fungus *Aspergillus* sp. SF-5280. Nat. Prod. Res..

[60] Kaur A., Raja H.A., Darveaux B.A., Chen W.L., Swanson S.M., Pearce C.J., Oberlies N.H. (2015). New diketopiperazine dimer from a filamentous fungal isolate of *Aspergillus sydowii*. Magn. Reson. Chem..

[61] Ovenden S.P., Sberna G., Tait R.M., Wildman H.G., Patel R., Li B., Steffy K., Nguyen N., Meurer-Grimes B.M. (2004). A diketopiperazine dimer from a marine-derived isolate of *Aspergillus niger*. J. Nat. Prod..

[62] Pérez-Balado C., Rodríguez-Graña P., de Lera A.R. (2009). Stereocontrolled and versatile total synthesis of bispyrrolidinoindoline diketopiperazine alkaloids: Structural revision of the fungal isolate (+)-asperdimin. Chemistry.

[63] Gomes N.M., Dethoup T., Singburaudom N., Gales L., Silva A.M.S., Kijjoa A. (2012). Eurocristatine, a new diketopiperazine dimer from the marine sponge-associated fungus *Eurotium cristatum*. Phytochem. Lett..

[64] Li Y., Sun K.L., Wang Y., Fu P., Liu P.P., Wang C., Zhu W.M. (2013). A cytotoxic pyrrolidinoindoline diketopiperazine dimer from the algal fungus *Eurotium herbariorum* HT-2. Chin. Chem. Lett..

[65] May Zin W.W., Buttachon S., Dethoup T., Pereira J.A., Gales L., Inácio Â., Costa P.M., Lee M., Sekeroglu N., Silva A.M.S. (2017). Antibacterial and antibiofilm activities of the metabolites isolated from the culture of the mangrove-derived endophytic fungus *Eurotium chevalieri* KUFA 0006. Phytochemistry.

[66] Tadano S., Sugimachi Y., Sumimoto M., Tsukamoto S., Ishikawa H. (2016). Collective synthesis and biological evaluation of tryptophan-based dimeric diketopiperazine alkaloids. Chemistry.

[67] Du F.Y., Li X.M., Li C.S., Shang Z., Wang B.G. (2012). Cristatumins A-D, new indole alkaloids from the marine-derived endophytic fungus *Eurotium cristatum* EN-220. Bioorg. Med. Chem. Lett..

[68] Lorenzo P., Álvarez R., de Lera Á.R. (2014). Total synthesis and structural revision of (+)-cristatumin C. J. Nat. Prod..

[69] Liu J., Gu B., Yang L., Yang F., Lin H. (2018). New anti-inflammatory cyclopeptides from a sponge-derived fungus *Aspergillus violaceofuscus*. Front. Chem..

[70] Weindling R., Emerson O.H. (1936). The isolation of a toxic substance from the culture filtrate of *Trichoderma*. Phytopathology.

[71] Bell M.R., Johnson J.R., Wildi B.S., Woodward R.B. (1958). The structure of gliotoxin. J. Am. Chem. Soc..

[72] Gardiner M.D., Waring P., Howlett B.J. (2005). The epipolythiodioxopiperazine (ETP) class of fungal toxins: Distribution, mode of action, functions and biosynthesis. Microbiology.

[73] Welch T.R., Williams R.M. (2014). Epidithiodioxopiperazines: Occurrence, synthesis and biogenesis. Nat. Prod. Rep..

[74] Chai C.L., Waring P. (2000). Redox sensitive epidithiodioxopiperazines in biological mechanisms of toxicity. Redox Rep..

[75] Waring P., Chai C.L.L. (2015). The multiple properties of gliotoxin and other epipolythiodioxopiperazine metabolites. Aust. J. Chem..

[76] Iwasa E., Hamashima Y., Sodeoka M. (2011). Epipolythiodiketopiperazine alkaloids: Total syntheses and biological activities. Isr. J. Chem..

[77] Jiang C.S., Guo Y.W. (2011). Epipolythiodioxopiperazines from fungi: Chemistry and bioactivities. Mini Rev. Med. Chem..

[78] Son B.W., Jensen P.R., Kauffman C.A., Fenical W. (1999). New cytotoxic epidithiodioxopiperazines related to verticillin A from a marine isolate of the fungus *Penicillium*. Nat. Prod. Lett..

[79] Figueroa M., Graf T.N., Ayers S., Adcock A.F., Kroll D.J., Yang J., Swanson S.M., Munoz-Acuna U., Carcache de Blanco E.J., Agrawal R. (2012). Cytotoxic epipolythiodioxopiperazine alkaloids from filamentous fungi of the Bionectriaceae. J. Antibiot..

[80] Dong J.Y., He H.P., Shen Y.M., Zhang K.Q. (2005). Nematicidal epipolysulfanyldioxopiperazines from *Gliocladium roseum*. J. Nat. Prod..

[81] Katagiri K., Sato K., Hayakawa S., Matsushima T., Minato H. (1970). Verticillin A, a new antibiotic from *Verticillium* sp.. J. Antibiot..

[82] Minato H., Matsumoto M., Katayama T. (1971). Verticillin A, a new antibiotic from *Verticillium* sp.. J. Chem. Soc. D.

[83] Paschall A.V., Yang D., Lu C., Choi J.H., Li X., Liu F., Figueroa M., Oberlies N.H., Pearce C., Bollag W.B. (2015). H3K9 trimethylation silences Fas expression to confer colon carcinoma immune escape and 5-fluorouracil chemoresistance. J. Immunol..

[84] Lu C., Paschall A.V., Shi H., Savage N., Waller J.L., Sabbatini M.E., Oberlies N.H., Pearce C., Liu K. (2017). The MLL1-H3K4me3 axis-mediated PD-L1 expression and pancreatic cancer immune evasion. J. Natl. Cancer Inst..

[85] Chu M., Truumees I., Rothofsky M.L., Patel M.G., Gentile F., Das P.R., Puar M.S., Lin S.L. (1995). Inhibition of *c-fos* proto-oncogene induction by Sch 52900 and Sch 52901, novel diketopiperazine produced by *Gliocladium* sp.. J. Antibiot..

[86] Liu F., Liu Q., Yang D., Bollag W.B., Robertson K., Wu P., Liu K. (2011). Verticillin A overcomes apoptosis resistance in human colon carcinoma through DNA methylation-dependent upregulation of BNIP3. Cancer Res..

[87] Lu C., Yang D., Sabbatini M.E., Colby A.H., Grinstaff M.W., Oberlies N.H., Pearce C., Liu K. (2018). Contrasting roles of H3K4me3 and H3K9me3 in regulation of apoptosis and gemcitabine resistance in human pancreatic cancer cells. BMC Cancer.

[88] Zewdu A., Lopez G., Braggio D., Kenny C., Constantino D., Bid H.K., Batte K., Iwenofu O.H., Oberlies N.H., Pearce C.J. (2016). Verticillin A inhibits leiomyosarcoma and malignant peripheral nerve sheath tumor growth via induction of apoptosis. Clin. Exp. Pharmacol..

[89] Zhang Y.X., Chen Y., Guo X.N., Zhang X.W., Zhao W.M., Zhong L., Zhou J., Xi Y., Lin L.P., Ding J. (2005). 11,11′-dideoxy-verticillin: A natural compound possessing growth factor receptor tyrosine kinase-inhibitory effect with anti-tumor activity. Anticancer Drugs.

[90] Chen Y., Zhang Y.X., Li M.H., Zhao W.M., Shi Y.H., Miao Z.H., Zhang X.W., Lin L.P., Ding J. (2005). Antiangiogenic activity of 11,11′-dideoxyverticillin, a natural product isolated from the fungus *Shiraia bambusicola*. Biochem. Biophys. Res. Commun..

[91] Watts K.R., Ratnam J., Ang K.H., Tenney K., Compton J.E., McKerrow J., Crews P. (2010). Assessing the trypanocidal potential of natural and semisynthetic diketopiperazines from two deep water marine-derived fungi. Bioorg. Med. Chem..

[92] Ebrahim W., Kjer J., Amrani M.E., Wray V., Lin W., Ebel R., Lai D., Proksch P. (2012). Pullularins E and F, two new peptides from the endophytic fungus *Bionectria ochroleuca* isolated from the mangrove plant *Sonneratia caseolaris*. Mar. Drugs.

[93] Joshi B.K., Gloer J.B., Wicklow D.T. (1999). New verticillin and glisoprenin analogues from *Gliocladium catenulatum*, a mycoparasite of *Aspergillus flavus* sclerotia. J. Nat. Prod..

[94] Zheng C.J., Kim C.J., Bae K.S., Kim Y.H., Kim W.G. (2006). Bionectins A-C, epidithiodioxopiperazines with anti-MRSA activity, from *Bionectra byssicola* F120. J. Nat. Prod..

[95] Zheng C.J., Park S.H., Koshino H., Kim Y.H., Kim W.G. (2007). Verticillin G, a new antibacterial compound from *Bionectra byssicola*. J. Antibiot..

[96] Hauser D., Weber H.P., Sigg H.P. (1970). Isolierung and strukturaufklärung von Chaetocin. Helv. Chem. Acta.

[97] Weber H.P. (1972). The molecular structure and absolute configuration of chaetocin. Acta Crystallogr. Sect. B Struct. Sci..

[98] Greiner D., Bonaldi T., Eskeland R., Roemer E., Imhof A. (2005). Identification of a specific inhibitor of the histone methyltransferase SU(VAR)3–9. Nat. Chem. Biol..

[99] Greiner D., Bonaldi T., Eskeland R., Roemer E., Imhof A. (2013). Reply to “Chaetocin is a nonspecific inhibitor of histone lysine methyltransferases”. Nat. Chem. Biol..

[100] Cherblanc F.L., Chapman K.L., Reid J., Borg A.J., Sundriyal S., Alcazar-Fuoli L., Bignell E., Demetriades M., Schofield C.J., DiMaggio P.A. (2013). On the histone lysine methyltransferase activity of fungal metabolite chaetocin. J. Med. Chem..

[101] Sodeoka M., Dodo K., Teng Y., Iuchi K., Hamashima Y., Iwasa E., Fujishiro S. (2012). Synthesis and biological activities of chaetocin and its derivatives. Pure Appl. Chem..

[102] Tran H.T., Kim H.N., Lee I.K., Nguyen-Pham T.N., Ahn J.S., Kim Y.K., Lee J.J., Park K.S., Kook H., Kim H.J. (2013). Improved therapeutic effect against leukemia by a combination of the histone methyltransferase inhibitor chaetocin and the histone deacetylase inhibitor trichostatin A. J. Korean Med. Sci..

[103] Lai Y.S., Chen J.Y., Tsai H.J., Chen T.Y., Hung W.C. (2015). The SUV39H1 inhibitor chaetocin induces differentiation and shows synergistic cytotoxicity with other epigenetic drugs in acute myeloid leukemia cells. Blood Cancer J..

[104] Liu X., Guo S., Liu X., Su L. (2015). Chaetocin induces endoplasmic reticulum stress response and leads to death receptor 5-dependent apoptosis in human non-small cell lung cancer cells. Apoptosis.

[105] Mathison A., Salmonson A., Missfeldt M., Bintz J., Williams M., Kossak S., Nair A., de Assunção T.M., Christensen T., Buttar N. (2017). Combined AURKA and H3K9 methyltransferase targeting inhibits cell growth by inducing mitotic catastrophe. Mol. Cancer Res..

[106] Isham C.R., Tibodeau J.D., Bossou A.R., Merchan J.R., Bible K.C. (2012). The anticancer effects of chaetocin are independent of programmed cell death and hypoxia, and are associated with inhibition of endothelial cell proliferation. Br. J. Cancer.

[107] Jung H.J., Seo I., Casciello F., Jacquelin S., Lane S.W., Suh S.I., Suh M.H., Lee J.S., Baek W.K. (2016). The anticancer effect of chaetocin is enhanced by inhibition of autophagy. Cell Death Dis..

[108] Chaib H., Nebbioso A., Prebet T., Castellano R., Garbit S., Restouin A., Vey N., Altucci L., Collette Y. (2012). Anti-leukemia activity of chaetocin via death receptor-dependent apoptosis and dual modulation of the histone methyl-transferase SUV39H1. Leukemia.

[109] Tibodeau J.D., Benson L.M., Isham C.R., Owen W.G., Bible K.C. (2009). The anticancer agent chaetocin is a competitive substrate and inhibitor of thioredoxin reductase. Antioxid. Redox Signal..

[110] Isham C.R., Tibodeau J.D., Jin W., Xu R., Timm M.M., Bible K.C. (2007). Chaetocin: A promising new antimyeloma agent with in vitro and in vivo activity mediated via imposition of oxidative stress. Blood.

[111] Han X., Han Y., Zheng Y., Sun Q., Ma T., Zhang J., Xu L. (2017). Chaetocin induces apoptosis in human melanoma cells through the generation of reactive oxygen species and the intrinsic mitochondrial pathway, and exerts its antitumor activity *in vivo*. PLoS ONE.

[112] Dixit D., Ghildiyal R., Anto N.P., Sen E. (2014). Chaetocin-induced ROS-mediated apoptosis involves ATM-YAP1 axis and JNK-dependent inhibition of glucose metabolism. Cell. Death Dis..

[113] Li Z., Huang L., Wei L., Hou Z., Ye W., Huang S. (2019). Chaetocin induces caspase-dependent apoptosis in ovarian cancer cells via the generation of reactive oxygen species. Oncol. Lett..

[114] He J., Chen X., Li B., Zhou W., Xiao J., He K., Zhang J., Xiang G. (2017). Chaetocin induces cell cycle arrest and apoptosis by regulating the ROS-mediated ASK-1/JNK signaling pathways. Oncol. Rep..

[115] Yang G., Weng X., Zhao Y., Zhang X., Hu Y., Dai X., Liang P., Wang P., Ma L., Sun X. (2017). The histone H3K9 methyltransferase SUV39H links SIRT1 repression to myocardial infarction. Nat. Commun..

[116] Kung A.L., Zabludoff S.D., France D.S., Freedman S.J., Tanner E.A., Vieira A., Cornell-Kennon S., Lee J., Wang B., Wang J. (2004). Small molecule blockade of transcriptional coactivation of the hypoxia-inducible factor pathway. Cancer Cell.

[117] Lee Y.M., Lim J.H., Yoon H., Chun Y.S., Park J.W. (2011). Antihepatoma activity of chaetocin due to deregulated splicing of hypoxia-inducible factor 1alpha pre-mRNA in mice and in vitro. Hepatology.

[118] Reece K.M., Richardson E.D., Cook K.M., Campbell T.J., Pisle S.T., Holly A.J., Venzon D.J., Liewehr D.J., Chau C.H., Price D.K. (2014). Epidithiodiketopiperazines (ETPs) exhibit in vitro antiangiogenic and in vivo antitumor activity by disrupting the HIF-1α/p300 complex in a preclinical model of prostate cancer. Mol. Cancer.

[119] Vo M.C., Nguyen-Pham T.N., Lee H.J., Jung S.H., Choi N.R., Hoang M.D., Kim H.J., Lee J.J. (2017). Chaetocin enhances dendritic cell function via the induction of heat shock protein and cancer testis antigens in myeloma cells. Oncotarget.

[120] Ono T., Kamimura N., Matsuhashi T., Nagai T., Nishiyama T., Endo J., Hishiki T., Nakanishi T., Shimizu N., Tanaka H. (2017). The histone 3 lysine 9 methyltransferase inhibitor chaetocin improves prognosis in a rat model of high salt diet-induced heart failure. Sci. Rep..

[121] Zuma A.A., Santos J.O., Mendes I., de Souza W., Machado C.R., Motta M.C.M. (2017). Chaetocin-A histone methyltransferase inhibitor-impairs proliferation, arrests cell cycle and induces nucleolar disassembly in *Trypanosoma cruzi*. Acta Trop..

[122] Bae J.S., Han M., Yao C., Chung J.H. (2016). Chaetocin inhibits IBMX-induced melanogenesis in B16F10 mouse melanoma cells through activation of ERK. Chem. Biol. Interact..

[123] Furuya K., Okudaira M., Shindo T., Sato A. (1985). *Corollospora pulchella*, a marine fungus producing antibiotics, melinacidins III, IV and gancidin W. Annu. Rep. Sankyo Res. Lab..

[124] Argoudelis A.D., Mizsak S.A. (1977). Melinacidins II, III and IV. Structural studies. J. Antibiot..

[125] Ebead G.A., Overy D.P., Berrué F., Kerr R.G. (2012). *Westerdykella reniformis* sp. nov., producing the antibiotic metabolites melinacidin IV and chetracin B. IMA Fungus.

[126] Li L., Li D., Luan Y., Gu Q., Zhu T. (2012). Cytotoxic metabolites from the Antarctic psychrophilic fungus *Oidiodendron truncatum*. J. Nat. Prod..

[127] Yu G., Wang Y., Yu R., Feng Y., Wang L., Che Q., Gu Q., Li D., Li J., Zhu T. (2018). Chetracins E and F, cytotoxic epipolythiodioxopiperazines from the marine-derived fungus *Acrostalagmus luteoalbus* HDN13–530. RSC Adv..

[128] Song X., Zhao Z., Qi X., Tang S., Wang Q., Zhu T., Gu Q., Liu M., Li J. (2015). Identification of epipolythiodioxopiperazines HDN-1 and chaetocin as novel inhibitor of heat shock protein 90. Oncotarget.

[129] Takahashi C., Numata A., Ito Y., Matsumura E., Araki H., Iwaki H., Kushida K. (1994). Leptosins, antitumour metabolites of a fungus isolated from a marine alga. J. Chem. Soc. Perkin Trans. 1.

[130] Takahashi C., Numata A., Matsumura E., Minoura K., Eto H., Shingu T., Ito T., Hasegawa T. (1994). Leptosins I and J, cytotoxic substances produced by a *Leptosphaeria* sp. Physico-chemical properties and structures. J. Antibiot..

[131] Takahashi C., Takai Y., Kimura Y., Numata A., Shigematsu N., Tanaka H. (1995). Cytotoxic metabolites from a fungal adherent of a marine alga. Phytochemistry.

[132] Takahashi C., Minoura K., Yamada T., Numata A., Kushida K., Shingu T., Hagishita S., Nakai H., Sato T., Harada H. (1995). Potent cytotoxic metabolites from a *Leptosphaeria* species. Structure determination and conformational analysis. Tetrahedron.

[133] Yamada T., Iwamoto C., Yamagaki N., Yamanouchi T., Minoura K., Yamori T., Uehara Y., Andoh T., Umemura K., Numata A. (2002). Leptosins M-N_1_, cytotoxic metabolites from a *Leptosphaeria* species separated from a marine alga. Structure determination and biological activities. Tetrahedron.

[134] Yamada T., Iwamoto C., Yamagaki N., Yamanouchi T., Minoura K., Hagishita S., Numata A. (2004). Leptosins O-S, cytotoxic metabolites from a strain of *Leptosphaeria* sp. isolated from a marine alga. Heterocycles.

[135] Du L., Robles A.J., King J.B., Mooberry S.L., Cichewicz R.H. (2014). Cytotoxic dimeric epipolythiodiketopiperazines from the Ascomycetous fungus *Preussia typharum*. J. Nat. Prod..

[136] Yanagihara M., Sasaki-Takahashi N., Suguhara T., Yamamoto S., Shinomi M., Yamashita I., Hayashida M., Yamanoha B., Numata A., Yamori T. (2005). Leptosins isolated from marine fungus *Leptoshaeria* species inhibit DNA topoisomerases I and/or II and induce apoptosis by inactivation of Akt/protein kinase B. Cancer Sci..

[137] Raju R., Piggott A.M., Conte M., Aalbersberg W.G., Feussner K., Capon R.J. (2009). Naseseazines A and B: A new dimeric diketopiperazine framework from a marine-derived Actinomycete, *Streptomyces* sp.. Org. Lett..

[138] Kim J., Movassaghi M. (2011). Concise total synthesis and stereochemical revision of (+)-naseseazines A and B: Regioselective arylative dimerization of diketopiperazine alkaloids. J. Am. Chem. Soc..

[139] Buedenbender L., Grkovic T., Duffy S., Kurtböke D.I., Avery V.M., Carroll A.R. (2016). Naseseazine C, a new anti-plasmodial dimeric diketopiperazine from a marine sediment derived *Streptomyces* sp.. Tetrahedron Lett..

[140] Xiong Z.Q., Liu Q.X., Pan Z.L., Zhao N., Feng Z.X., Wang Y. (2015). Diversity and bioprospecting of culturable actinomycetes from marine sediment of the Yellow Sea, China. Arch. Microbiol..

[141] Tian W., Sun C., Zheng M., Harmer J.R., Yu M., Zhang Y., Peng H., Zhu D., Deng Z., Chen S.L. (2018). Efficient biosynthesis of heterodimeric C^3^-aryl pyrroloindoline alkaloids. Nat. Commun..

[142] Varoglu M., Corbett T.H., Valeriote F.A., Crews P. (1997). Asperazine, a selective cytotoxic alkaloid from a sponge-derived culture of *Aspergillus niger*. J. Org. Chem..

[143] Varoglu M., Crews P. (2000). Biosynthetically diverse compounds from a saltwater culture of sponge-derived *Aspergillus niger*. J. Nat. Prod..

[144] Xu B., Zou K., Cheng F. (2014). Alkaloids from *Penicillium oxalicum*, a fungus residing in *Acrida cinerea*. Adv. Mater. Res..

[145] Govek S.P., Overman L.E. (2007). Total synthesis of (+)-asperazine. Tetrahedron.

[146] Ding G., Jiang L., Guo L., Chen X., Zhang H., Che Y. (2008). Pestalazines and pestalamides, bioactive metabolites from the plant pathogenic fungus *Pestalotiopsis theae*. J. Nat. Prod..

[147] Zulqarnain, Iqbal Z., Cox R., Anwar J., Ahmad N., Khan K., Iqbal M., Manzoor N., Nhattak S.U. (2018). Antifungal activity of compounds isolated from *Aspergillus niger* and their molecular docking studies with tomatinase. Nat. Prod. Res..

[148] Xiao J., Zhang Q., Gao Y.Q., Shi X.W., Gao J.M. (2014). Antifungal and antibacterial metabolites from an endophytic *Aspergillus* sp. associated with *Melia azedarach*. Nat. Prod. Res..

[149] Cai S., Kong X., Wang W., Zhou H., Zhu T., Li D., Gu Q. (2012). Aspergilazine A, a diketopiperazine dimer with a rare N-1 to C-6 linkage, from a marine-derived fungus *Aspergillus taichungensis*. Tetrahedron Lett..

[150] Yun K., Khong T.T., Leutou A.S., Kim G.D., Hong J., Lee C.H., Son B.W. (2016). Cristazine, a new cytotoxic dioxopiperazine alkaloid from the mudflat-sediment-derived fungus *Chaetomium cristatum*. Chem. Pharm. Bull..

[151] Geiger W.B. (1949). Chetomin an antibiotic substance from *Chaetomium cochliodes*. Arch. Biochem..

[152] Waksman S.A., Bugie E. (1944). Chaetomin, a new antibiotic substance produced by *Chaetomium cochliodes*. J. Bact..

[153] Brewer D., Duncan J.M., Jerram W.A., Leach C.K., Safe S., Taylor A., Vining L.C., Archibald R.M., Stevenson R.G., Mirocha C.J. (1972). Ovine ill-thrift in Nova Scotia. 5. The production and toxicology of chetomin, a metabolite of *chaetomium* spp.. Can. J. Microbiol..

[154] Brewer D., McInnes A.G., Smith D.G., Taylor A., Walter J.A., Loosli H.R., Kis Z.L. (1987). Sporidesmins. Part 16. The structure of chetomin, a toxic metabolite of *Chaetomium cochliodes*, by nitrogen-15 and carbon-13 nuclear magnetic resonance spectroscopy. J. Chem. Soc. Perkin Trans. 1.

[155] McInnes A.G., Taylor A., Walter J.A. (1976). The structure of chetomin. J. Am. Chem. Soc..

[156] Safe S., Taylor A. (1972). Sporidesmins. Part XIII. Ovine Ill-thrift in Nova Scotia. Part III. The characterisation of chetomin a toxic metabolite of *Chaetomium cochliodes* and *Chaetomium globosum*. J. Chem. Soc. Perkin Trans. 1.

[157] Kikuchi T., Kadota S., Nakamura K., Nishi A., Taga T., Kaji T., Osaki K., Tubaki K. (1982). Dethio-tetra(methylthio)chetomin, a new antimicrobial metabolite of *Chaetomium globosum* KINZE ex FR. Structure and partial synthesis from chetomin. Chem. Pharm. Bull..

[158] Brewer D., Calder F.W., MacIntyre T.M., Taylor A. (1971). Ovine ill-thrift in Nova Scotia: I. The possible regulation of the rumen flora in sheep by the fungal flora of permanent pasture. J. Agric. Sci..

[159] Brewer D., Hannah D.E., Taylor A. (1966). The biological properties of 3,6-epidithiadiketopiperazines: Inhibition of growth of *Bacills subtilis* by gliotoxins, sporidesmins, and chetomin. Can. J. Microbiol..

[160] Brewer D., Hannah D.E., Rahman R., Taylor A. (1967). The growth of *Bacillus subtilis* in media containing chetomin, sporidesmin, and gliotoxin. Can. J. Microbiol..

[161] Jen W.C., Jones G.A. (1983). Effects of chetomin on growth and acidic fermentation products of rumen bacteria. Can. J. Microbiol..

[162] Cook K.M., Hilton S.T., Mecinović J., Motherwell W.B., Figg W.D., Schofield C.J. (2009). Epidithiodiketopiperazines block the interaction between hypoxia-inducible factor-1α (HIF-1α) and p300 by a zinc ejection mechanism. J. Biol. Chem..

[163] Kessler J., Hahnel A., Wichmann H., Rot S., Kappler M., Bache M., Vordemark D. (2010). HIF-1α inhibition by siRNA or chetomin in human malignant glioma cells: Effects on hypoxic radioresistance and monitoring via CA9 expression. BMC Cancer.

[164] Horiuchi A., Hayashi T., Kikuchi N., Hayashi A., Fuseya C., Shiozawa T., Konishi I. (2012). Hypoxia upregulates ovarian cancer invasiveness via the binding of HIF-1alpha to a hypoxia-induced, methylation-free hypoxia response element of S100A4 gene. Int. J. Cancer.

[165] Viziteu E., Grandmougin C., Goldschmidt H., Seckinger A., Hose D., Klein B., Moreaux J. (2016). Chetomin, targeting HIF-1α/p300 complex, exhibits antitumour activity in multiple myeloma. Br. J. Cancer.

[166] Staab A., Loeffler J., Said H.M., Diehlmann D., Katzer A., Beyer M., Fleischer M., Schwab F., Baier K., Einsele H. (2007). Effects of HIF-1 inhibition by chetomin on hypoxia-related transcription and radiosensitivity in HT 1080 human fibrosarcoma cells. BMC Cancer.

[167] Indelicato M., Pucci B., Schito L., Reali V., Aventaggiato M., Mazzarino M.C., Stivala F., Fini M., Russo M.A., Tafani M. (2010). Role of hypoxya and autophagy in MDA-MB-231 invasiveness. J. Cell Physiol..

[168] Dewangan J., Srivastava S., Mishra S., Pandey P.K., Divakar A., Rath S.K. (2018). Chetomin induces apoptosis in human triple-negative breast cancer cells by promoting calcium overload and mitochondrial dysfunction. Biochem. Biophys. Res. Commun..

[169] Hiraki M., Hwang S.Y., Cao S., Ramadhar T.R., Byun S., Yoon K.W., Lee J.H., Chu K., Gurkar A.U., Kolev V. (2015). Small-molecule reactivation of mutant p53 to wild-type-like p53 through the p53-Hsp40 regulatory axis. Chem. Biol..

[170] Yano K., Horinaka M., Yoshida T., Yasuda T., Taniguchi H., Goda A.E., Wakada M., Yoshikawa S., Nakamura T., Kawauchi A. (2011). Chetomin induces degradation of XIAP and enhances TRAIL sensitivity in urogenital cancer cells. Int. J. Oncol..

[171] Takahashi M., Takemoto Y., Shimazu T., Kawasaki H., Tachibana M., Shinkai Y., Takagi M., Shin-ya K., Igarashi Y., Ito A. (2012). Inhibition of histone H3K9 methyltransferases by gliotoxin and related epipolythiodioxopiperazines. J. Antibiot..

[172] Hwang I.I., Watson I.R., Der S.D., Ohh M. (2006). Loss of VHL confers hypoxia-inducible factor (HIF)-dependent resistance to vesicular stomatitis virus: Role of HIF in antiviral response. J. Virol..

[173] Fujimoto H., Sumino M., Okuyama E., Ishibashi M. (2004). Immunomodulatory constituents from an Ascomycete, *Chaetomium seminudum*. J. Nat. Prod..

[174] Herath K.B., Jayasuriya H., Ondeyka J.G., Polishook J.D., Bills G.F., Dombrowski A.W., Cabello A., Vicario P.P., Zweerink H., Guan Z. (2005). Isolation and structures of novel fungal metabolites as chemokine receptor (CCR2) antagonists. J. Antibiot..

[175] Welch T.R., Williams R.M. (2013). Studies on the biosynthesis of chetomin: Enantiospecific synthesis of a putative, late-stage biosynthetic intermediate. Tetrahedron.

[176] Jo M.J., Patil M.P., Jung H.I., Seo Y.B., Lim H.K., Son B.W., Kim G.D. (2019). Cristazine, a novel dioxopiperazine alkaloid, induces apoptosis via the death receptor pathway in A431 cells. Drug Dev. Res..

[177] Song F., Liu X., Guo H., Ren B., Chen C., Piggott A.M., Yu K., Gao H., Wang Q., Liu M. (2012). Brevianamides with antitubercular potential from a marine-derived isolate of *Aspergillus versicolor*. Org. Lett..

[178] Anjaneyulu M., Gopal N., Rao A. (1996). A novel dimeric dipeptide from the Indian-Ocean starfish *Pentaceraster regulus*. J. Chem. Res. Synop..

[179] Giessen T.B., Marahiel M.A. (2015). Rational and combinatorial tailoring of bioactive cyclic dipeptides. Front. Microbiol..

[180] Koglin A., Walsh C.T. (2009). Structural insights into nonribosomal peptide enzymatic assembly lines. Nat. Prod. Rep..

[181] Yin W.B., Grundmann A., Cheng J., Li S.M. (2009). Acetylaszonalenin biosynthesis in *Neosartorya fischeri*. Identification of the biosynthetic gene cluster by genomic mining and functional proof of the genes by biochemical investigation. J. Biol. Chem..

[182] Maiya S., Grundmann A., Li S.M., Turner G. (2006). The fumitremorgin gene cluster of *Aspergillus fumigatus*: Identification of a gene encoding brevianamide F synthetase. ChemBioChem.

[183] Schenke D., Böttcher C., Lee J., Scheel D. (2011). Verticillin A is likely not produced by *Verticillium* sp.. J. Antibiot..

[184] Costa M., Rosa F., Ribeiro T., Hernandez-Bautista R., Bonaldo M., Gonçalves Silva N., Eiríksson F., Thorsteinsdóttir M., Ussar S., Urbatzka R. (2019). Identification of cyanobacterial strains with potential for the treatment of obesity-related co-morbidities by bioactivity, toxicity evaluation and metabolite profiling. Mar. Drugs.

[185] Overy D.P., Berrue F., Correa H., Hanif N., Hay K., Lanteigne M., Mquilian K., Duffy S., Boland P., Jagannathan R. (2014). Sea foam as a source of fungal inoculum for the isolation of biologically active natural products. Mycology.

[186] Davidson B.S. (1995). New dimensions in natural products research: Cultured marine microorganisms. Curr. Opin. Biotechnol..

[187] Kobayashi J., Ishibashi M. (1993). Bioactive metabolites of symbiotic marine microorganisms. Chem. Rev..

[188] Schoch C.L., Crous P.W., Groenewald J.Z., Boehm E.W., Burgess T.I., de Gruyter J., de Hoog G.S., Dixon L.J., Grube M., Gueidan C. (2009). A class-wide phylogenetic assessment of *Dothideomycetes*. Stud. Mycol..

[189] Overy D.P., Bayman P., Kerr R.G., Bills G.F. (2014). An assessment of natural product discovery from marine (*sensu strictu*) and marine-derived fungi. Mycology.

[190] Hoffmeister D., Keller N.P. (2007). Natural products of filamentous fungi: Enzymes, genes, and their regulation. Nat. Prod. Rep..

[191] Amend A., Burgaud G., Cunliffe M., Edgcomb V.P., Ettinger C.L., Gutiérrez M.H., Heitman J., Hom E.F.Y., Ianiri G., Jones A.C. (2019). Fungi in the marine environment: Open questions and unsolved problems. MBio.

[192] Canu N., Belin P., Thai R., Correia I., Lequin O., Seguin J., Moutiez M., Gondry M. (2018). Incorporation of non-canonical amino acids into 2,5-diketopiperazines by cyclodipeptide synthases. Angew. Chem. Int. Ed. Engl..

[193] Rinke C., Schwientek P., Sczyrba A., Ivanova N.N., Anderson I.J., Cheng J.F., Darling A., Malfatti S., Swan B.K., Gies E.A. (2013). Insights into the phylogeny and coding potential of microbial dark matter. Nature.

[194] Walsh C.T. (2016). Insights into the chemical logic and enzymatic machinery of NRPS assembly lines. Nat. Prod. Rep..

[195] Spiteller P. (2015). Chemical ecology of fungi. Nat. Prod. Rep..

[196] Degrassi G., Aguilar C., Bosco M., Zahariev S., Pongor S., Venturi V. (2002). Plant growth-promoting *Pseudomonas putida* WCS358 produces and secretes four cyclic dipeptides: Cross-talk with quorum sensing bacterial sensors. Curr. Microbiol..

[197] Holden M.T., Ram Chhabra S., de Nys R., Stead P., Bainton N.J., Hill P.J., Manefield M., Kumar N., Labatte M., England D. (1999). Quorum-sensing cross talk: Isolation and chemical characterization of cyclic dipeptides from *Pseudomonas aeruginosa* and other Gram-negative bacteria. Mol. Microbiol..

[198] Park D.K., Lee K.E., Baek C.H., Kim I.H., Kwon J.H., Lee W.K., Lee K.H., Kim B.S., Choi S.H., Kim K.S. (2006). Cyclo(Phe-Pro) modulates the expression of *ompU* in *Vibrio* spp.. J. Bacteriol..

[199] Gardiner D.M., Cozijnsen A.J., Wilson L.M., Pedras M.S., Howlett B.J. (2004). The sirodesmin biosynthetic gene cluster of the plant pathogenic fungus *Leptosphaeria maculans*. Mol. Microbiol..

[200] Li J., Wang W., Xu S.X., Magarvey N.A., McCormick J.K. (2011). *Lactobacillus reuteri*-produced cyclic dipeptides quench *agr*-mediated expression of toxic shock syndrome toxin-1 in staphylococci. Proc. Natl. Acad. Sci. USA.

[201] Ortiz-Castro R., Diaz-Perez C., Martinez-Trujillo M., Del Rio R.E., Campos-Garcia J., Lopez-Bucio J. (2011). Trans kingdom signaling based on bacterial cyclodipeptides with auxin activity in plants. Proc. Natl. Acad. Sci. USA.

[202] Orr J.G., Leel V., Cameron G.A., Marek C.J., Haughton E.L., Erick L.J., Trim J.E., Hawksworth G.M., Halestrap A.P., Wright M.C. (2004). Mechanism of action of the antifibrogenic compound gliotoxin in rat liver cells. Hepatology.

[203] Debatin K.M., Poncet D., Kromer G. (2002). Chemotherapy: Targeting the mitochondrial death pathway. Oncogene.

[204] Sohtome Y., Sodeoka M. (2018). Development of chaetocin and *S*-adenosylmethionine analogues as tools for studying protein methylation. Chem. Rec..

[205] Fujishiro S., Dodo K., Iwasa E., Teng Y., Sohtome Y., Hamashima Y., Ito A., Yoshida M., Sodeoka M. (2013). Epidithiodiketopiperazine as a pharmacophore for protein lysine methyltransferase G9a inhibitors: Reducing cytotoxicity by structural simplification. Bioorg. Med. Chem. Lett..

[206] Jiang D.S., Fang Z., Zhu X.H., Wei X. (2017). The promising therapeutic agents for heart diseases: Histone Methyltransferase inhibitors. Int. J. Cardiol..

[207] Ciarkowski J. (1984). CNDO/2 quantum-mechanical calculations of the conformational flexibility of the diketopiperazine skeleton. Biopolymers.

[208] Liskamp R.M.J., Rijkers D.T.S., Kruijtzer J.A.W., Kemmink J. (2011). Peptides and proteins as a continuing exciting source of inspiration for peptidomimetics. ChemBioChem.

[209] Ray J.G., Ly J.T., Savin D.A. (2011). Peptide-based lipid mimetics with tunable core properties *via* thiol–alkyne chemistry. Polym. Chem..

[210] Hadden M.K., Blagg B.S.J. (2008). Dimeric approaches to anti-cancer chemotherapeutics. Anticancer Agents Med. Chem..

[211] Jervis P.J., Moulis M., Jukes J.-P., Ghadbane H., Cox L.R., Cerundolo V., Besra G.S. (2012). Towards multivalent CD1d ligands: Synthesis and biological activity of homodimeric α-galactosyl ceramide analogues. Carbohydr. Res..

[212] Lian G., Yu B. (2010). Naturally occurring dimers from chemical perspective. Chem. Biodivers..

[213] MacDonald S.J., Watson K.G., Cameron R., Chalmers D.K., Demaine D.A., Fenton R.J., Gower D., Hamblin J.N., Hamilton S., Hart G.J. (2004). Potent and long-acting dimeric inhibitors of influenza virus neuraminidase are effective at a once-weekly dosing regimen. Antimicrob. Agents Chemother..

[214] Muñoz-Torrero D., Camps P. (2006). Dimeric and hybrid anti-Alzheimer drug candidates. Curr. Med. Chem..

[215] Ni F., Kota S., Takahashi V., Strosberg A.D., Snyder J.K. (2011). Potent inhibitors of hepatitis C core dimerization as new leads for anti-hepatitis C agents. Bioorg. Med. Chem..

[216] Raspaglio G., Ferlini C., Mozzetti S., Prislei S., Gallo D., Das N., Scambia G. (2005). Thiocolchicine dimers: A novel class of topoisomerase-I inhibitors. Biochem. Pharm..

[217] Gomes N.G.M., Dasari R., Chandra S., Kiss R., Kornienko A. (2016). Marine invertebrate metabolites with anticancer activities: Solutions to the “supply problem”. Mar. Drugs.

[218] Kim J., Ashenhurst J.A., Movassaghi M. (2009). Total synthesis of (+)-11,11’-dideoxyverticillin A. Science.

[219] Boyd E.M., Sperry J. (2014). Total synthesis of (–)-aspergilazine A. Org. Lett..

[220] Liang K., Deng X., Tong X., Li D., Ding M., Zhou A., Xia C. (2015). Copper-mediated dimerization to access 3a,3a’-bispyrrolidinoindoline: Diastereoselective synthesis of (+)-WIN 64821 and (–)-ditryptophenaline. Org. Lett..

[221] Loach R.P., Fenton O.S., Movassaghi M. (2016). Concise total synthesis of (+)-asperazine, (+)-pestalazine A, and (+)-*iso*-pestalazine A. Structure revision of (+)-pestalazine A. J. Am. Chem. Soc..

[222] Tadano S., Mukaeda Y., Ishikawa H. (2013). Bio-inspired dimerization reaction of tryptophan derivatives in aqueous acidic media: Three-step syntheses of (+)-WIN 64821, (–)-ditryptophenaline, and (+)-naseseazine B. Angew. Chem. Int. Ed..

[223] Kim J., Movassaghi M. (2015). Biogenetically-inspired total synthesis of epidithiodiketopiperazines and related alkaloids. Acc. Chem. Res..

[224] Boyer N., Morrison K.C., Kim J., Hergenrother P.J., Movassaghi M. (2013). Synthesis and anticancer activity of epipolythiodiketopiperazine alkaloids. Chem. Sci..

[225] Iwasa E., Hamashima Y., Fujishiro S., Higuchi E., Ito A., Yoshida M., Sodeoka M. (2010). Total synthesis of (+)-chaetocin and its analogues: Their histone methyltransferase G9a inhibitory activity. J. Am. Chem. Soc..

[226] Teng Y., Iuchi K., Iwasa E., Fujishiro S., Hamashima Y., Dodo K., Sodeoka M. (2010). Unnatural enantiomer of chaetocin shows strong apoptosis-inducing activity through caspase-8/caspase-3 activation. Bioorg. Med. Chem. Lett..

[227] Wada M., Suzuki H., Kato M., Oikawa H., Tsubouchi A., Oguri H. (2019). Stereodivergent synthesis of bispyrrolidinoindoline alkaloidal scaffolds and generation of a lead candidate with stereospecific antiproliferative activity. ChemBioChem.

